# A mechanism of lysosomal calcium entry

**DOI:** 10.1126/sciadv.adk2317

**Published:** 2024-02-14

**Authors:** Matthew Zajac, Sourajit Mukherjee, Palapuravan Anees, Daphne Oettinger, Katharine Henn, Jainaha Srikumar, Junyi Zou, Anand Saminathan, Yamuna Krishnan

**Affiliations:** ^1^ Department of Chemistry, The University of Chicago, Chicago, IL 60637, USA.; ^2^ Neuroscience Institute, The University of Chicago, Chicago, IL 60637, USA.; ^3^ Institute for Biophysical Dynamics, The University of Chicago, Chicago, IL 60637, USA.

## Abstract

Lysosomal calcium (Ca^2+^) release is critical to cell signaling and is mediated by well-known lysosomal Ca^2+^ channels. Yet, how lysosomes refill their Ca^2+^ remains hitherto undescribed. Here, from an RNA interference screen in *Caenorhabditis elegans*, we identify an evolutionarily conserved gene, *lci-1*, that facilitates lysosomal Ca^2+^ entry in *C. elegans* and mammalian cells. We found that its human homolog TMEM165, previously designated as a Ca^2+^/H^+^ exchanger, imports Ca^2+^ pH dependently into lysosomes. Using two-ion mapping and electrophysiology, we show that TMEM165, hereafter referred to as human LCI, acts as a proton-activated, lysosomal Ca^2+^ importer. Defects in lysosomal Ca^2+^ channels cause several neurodegenerative diseases, and knowledge of lysosomal Ca^2+^ importers may provide previously unidentified avenues to explore the physiology of Ca^2+^ channels.

## Teaser

Human TMEM165 is a proton-activated, lysosomal Ca^2+^ importer.

## INTRODUCTION

Eukaryotic cells use several mechanisms to regulate the spatial and temporal dynamics of cytosolic Ca^2+^ (*1*). These include feedback with intracellular Ca^2+^ stores such as the endoplasmic reticulum (ER), lysosomes, or mitochondria, which have high Ca^2+^ content (*1*–*3*). These organelles harbor proteins that mediate Ca^2+^ release as well as import because after the organelle releases Ca^2+^ via an exporter, its lumenal Ca^2+^ must be replenished by a Ca^2+^ importer. Thus, after its release via the Ryanodine receptor in the ER, Ca^2+^ is refilled by SERCA (*4*, *5*), while in the mitochondria, after release via the Na^+^/Ca^2+^ exchanger, NCX, Ca^2+^ is replenished by the mitochondrial Ca^2+^ uniporter (MCU) (*6*–*9*). Lysosomes have recently come into prominence as the acidic Ca^2+^ stores of the cell (*10*) and while we know of many channels that release Ca^2+^ from lysosomes, no Ca^2+^ importers are known in humans (*11*).

Vacuoles perform the functions of lysosomes in plants and are known to harbor two kinds of Ca^2+^ importers: high-capacity Ca^2+^ exchangers and low-capacity Ca^2+^ adenosine triphosphatases (ATPases) (*12*, *13*). In mammalian lysosomes, both, a pH-dependent Ca^2+^ uptake mechanism and a Ca^2+^/H^+^ exchange mechanism, have been posited (*14*–*16*). The heterologous overexpression of Xenopus CAX in mammalian lysosomes leads to Ca^2+^ import (*17*), and a P-type Ca^2+^ ATPase, ATP13A2, facilitates Ca^2+^ accumulation (*18*). However, a molecule that directly transports Ca^2+^ into human lysosomes is yet to be described.

## RESULTS

### Targeted screen for potential lysosomal Ca^2+^ importers in nematodes

**Fig. 1.**
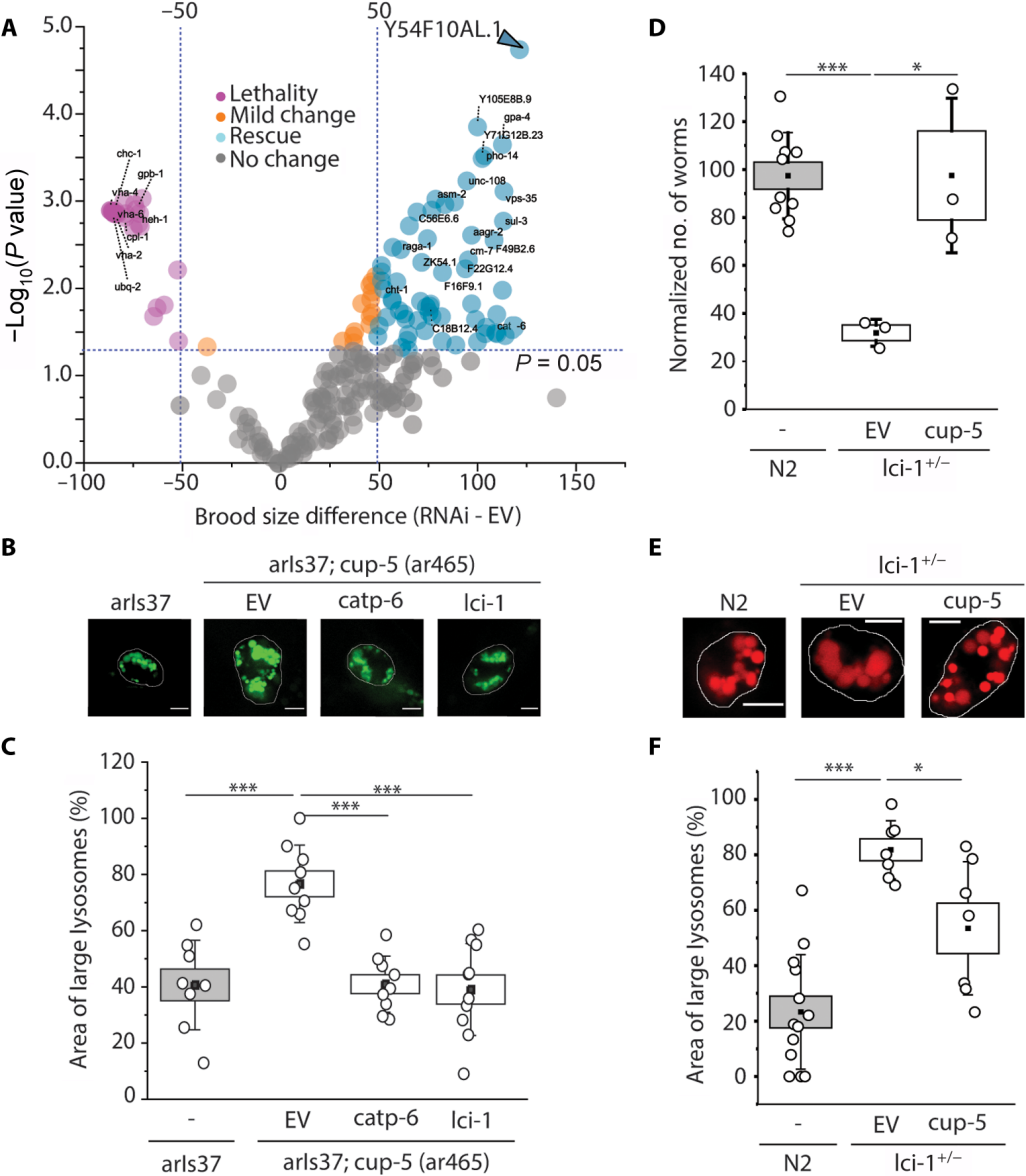
A phenotypic *sc*reen in *C. elegans* identifies *lci-1* as a facilitator of lysosomal Ca^2+^ import. (**A**) Survival of *cup-5*^+/−^ worms following RNAi knockdown of the 228 lysosomal genes, compared to worm survival following knockdown with an empty vector (EV). Genes increasing survival (rescue) have a brood size change >50 and *P* < 0.05, while genes decreasing survival (lethality) have a brood size change <−50 and *P* < 0.05. (**B**) Representative fluorescence images of lysosomes in coelomocytes of *arIs37* and *arIs37; cup-5(ar465)* worms following on RNAi knockdown of indicated proteins. (**C**) Percentage area occupied by enlarged lysosomes in the indicated genetic background (*n* > 10 cells, >75 lysosomes). (**D**) Number of N2 or *lci-1*^+/−^ progeny following RNAi knockdown of *cup-5*. (**E**) Representative fluorescence images of lysosomes in coelomocytes of *N2* and *lci-1^+/−^* worms in the indicated genetic background. Lysosomes are labeled with Alexa Fluor 647 duplex DNA. (**F**) Percentage area occupied by enlarged lysosomes in the indicated genetic background (*n* > 5 cells, >50 lysosomes). *ncx*, Na^+^/Ca^2+^ exchanger; *clh*-*6*, Cl^−^ channel protein; *catp-6*, Ca^2+^-transporting ATPase; *lci-1*, lysosomal Ca^2+^ importer. Scale bars, 5 μm. All experiments were performed in triplicate. Boxes and bars represent the SEM and SD, respectively. *P < 0.05; ***P < 0.001 [one-way analysis of variance (ANOVA) with Tukey post hoc test].

We performed a targeted RNA interference (RNAi) screen to identify lysosomal Ca^2+^ importers using assays that previously pinpointed ATP13A2 (CATP-6 in *Caenorhabditis elegans*), as facilitating import (*17*). Here, gene knockdown reverses the phenotypic defects in a lysosomal Ca^2+^ channel mutant (fig. S1A). In *C. elegans*, deleting *cup-5*, the worm homolog of the lysosomal Ca^2+^ release channel *MCOLN1*, is lethal (*19*). We knocked down 228 lysosomal genes in *cup-5*^+/−^ worms (table S1), screening for reversal of lethality, reasoning that a broken Ca^2+^ intake mechanism would reverse the phenotype. We found that knocking down the uncharacterized gene *Y54F10AL.1*, now denoted *lci-1*, in *cup-5*–deficient nematodes rescues lethality (Fig. 1A and fig. S1, B to D). Next, knocking down *cup-5* leads to abnormally large lysosomes due to storage arising from lysosome dysfunction. We therefore tested whether *lci-1* knockdown rescues the lysosomal *cup-5* phenotype. We used the *arIs37;cup-5(ar465)* strain because in this *cup-5* hypomorph, lysosomal dysfunction is severe enough to produce swollen, green fluorescent protein (GFP)–labeled lysosomes, yet is insufficient for lethality (*20*). Knocking down *lci-1* restores lysosome sizes to normalcy, indicating that lysosome function is restored potentially because aberrant lysosomal Ca^2+^ levels in *cup-5* defective nematodes are rebalanced (Fig. 1, B and C).

We then tested whether the relationship between *lci-1* and *cup-5* is commutative by seeing whether *cup-5* knockdown rescues *lci-1^−/−^* phenotypes. Homozygous *lci-1* knockout (KO) worms are embryonic lethal, *lci-1^+/−^* worms show small brood sizes, and their coelomocytes have swollen lysosomes (Fig. 1, D to F, and fig. S2, A to C). Knocking down *cup-5* in *lci-1^+/−^* worms rescues lethality and brood sizes (Fig. 1D and fig. S2D) and restores normal lysosome morphology (Fig. 1, E and F). Thus, *lci-1* acts in opposition to *cup-5*, likely facilitating lysosomal Ca^2+^ accumulation directly or indirectly.

### Human LCI facilitates lysosomal Ca^2+^ accumulation in nematodes

**Fig. 2.**
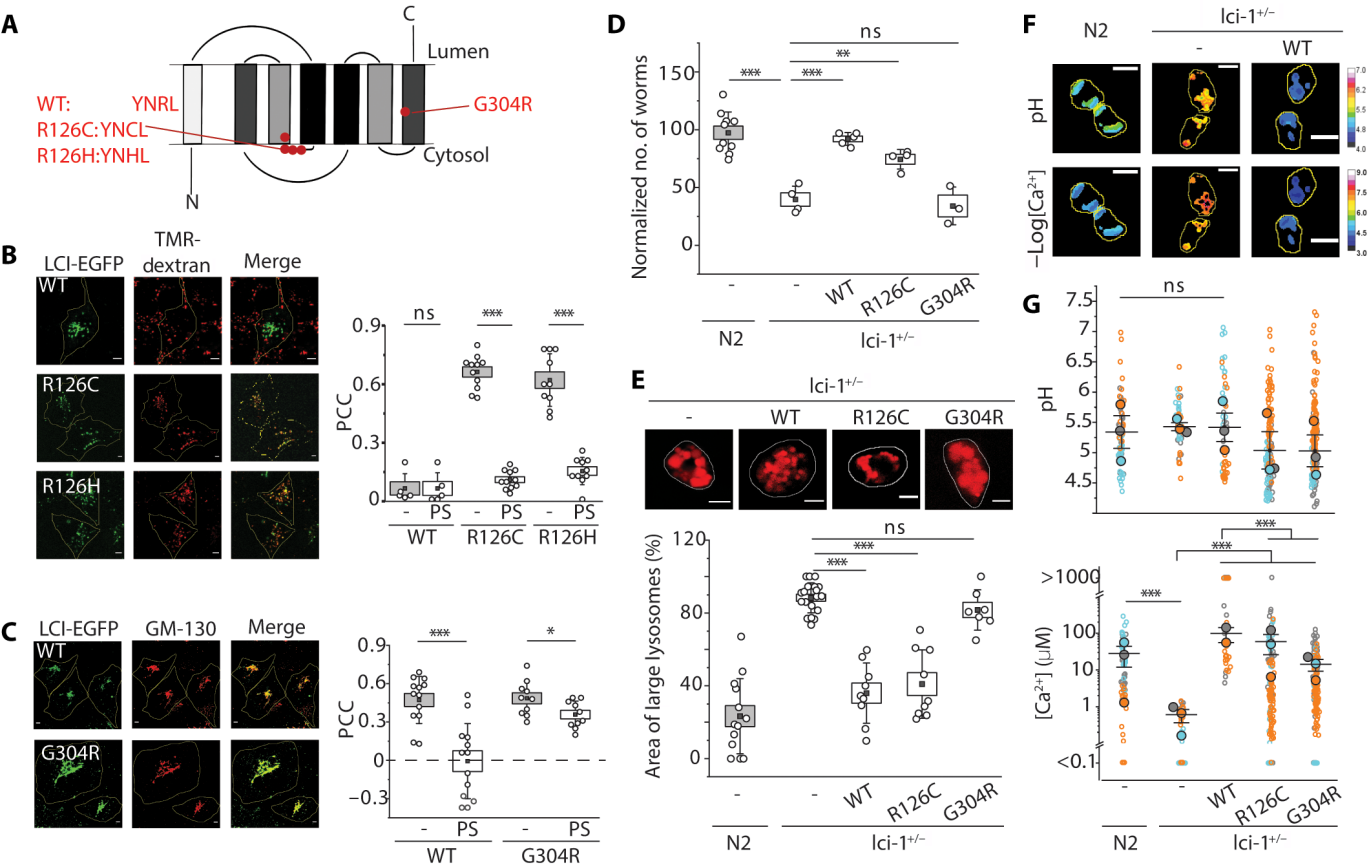
Human LCI rescues lysosomal Ca^2+^ import defects. (**A**) Schematic of human LCI topology showing internal symmetry of transmembrane helices and location of key mutations. (**B**) Left: Representative fluorescence images of COS-7 cells transiently expressing the indicated variant of human LCI-EGFP– (green) and TMR-dextran–labeled lysosomes (red). Right: Pearson’s correlation coefficient (PCC) of human LCI and TMR-dextran before and after pixel shift (PS). (**C**) Left: Representative fluorescence images of COS-7 cells transfected with the indicated variant of human LCI-EGFP (green) and immunostained for GM130 (red). Right: PCC of human LCI and GM130 before and after PS. *n* > 10 cells. (**D**) Number of the progeny of *lci-1*^+/−^ worms extrachromosomally expressing the indicated human LCI variant (*n* = 3 replicates). (**E**) Top: Representative fluorescence images of lysosomes in coelomocytes of worms in the indicated genetic background. Lysosomes are labeled with Alexa Fluor 647 duplex DNA. Bottom: Percentage of area occupied by enlarged lysosomes in worms of the indicated genetic background. *n* > 5 cells, >50 lysosomes. (**F**) Representative pH and –log([Ca^2+^]) maps in *CalipHluor2.0*-labeled lysosomes in coelomocytes in indicated genetic backgrounds. (**G**) Distribution of lysosomal pH (top) and lysosomal Ca^2+^ (bottom) in the indicated genetic backgrounds. Data represent the means (closed blue, orange, and gray circles) of three independent biological replicate sets of individual lysosomes (open blue, orange, and gray circles). Lysosomes with Ca^2+^ levels below O/R_min_ or above O/R_max_ of *CalipHluor2.0* are plotted as <0.1 and >1000 μM, respectively. Scale bars, 5 μm. Boxes and bars represent the SEM and SD, respectively. The I-shaped box represents the means ± SEM. ns, *P* > 0.05; *P < 0.05; **P < 0.01; ***P < 0.001 (one-way ANOVA with Tukey post hoc test). ns, not significant; WT, wild type.

The gene *lci-1* is evolutionarily conserved from yeast (*GDT1*) to humans (*TMEM165*) (*21*). The human homolog is implicated in selected congenital disorders of glycosylation (CDG). While TMEM165, hereafter denoted human LCI, is predominantly Golgi-resident, a moderate fraction is also present in lysosomes (*22*, *23*). Missense mutations in human LCI lead to it favoring either Golgi localization (compound heterozygous G304R and R126C) or lysosomal localization (homozygous R126C/H) (*24*). In humans, loss of LCI localization in either organelle causes growth and psychomotor retardation, muscular weakness, severe dwarfism, and death in infancy (*23*, *25*). R126 lies within a putative YNRL lysosomal-targeting motif (Fig. 2A) (*24*). In our hands too, human LCI-EGFP (enhanced green fluorescent protein) is Golgi-resident, the R126C or R126H variants are predominantly in lysosomes, and the G304R variant is predominantly in the Golgi (Fig. 2, B and C, text S1, and fig. S3). A DsRed fusion of wild-type (WT) human LCI and endogenous human LCI was clearly present on lysosomal membranes (fig. S4) (*26*), reaffirming that in addition to the Golgi, a small, yet notable, fraction resides in lysosomes.

We then tested whether human LCI can rescue *lci-1^+/−^* worm phenotypes, by extrachromosomally expressing either lysosome-favoring (R126C) or Golgi-favoring (G304R) human LCI variants in these worms (fig. S5A). Only WT human LCI and R126C human LCI rescued the brood size and lysosome morphology defects of *lci-1*^+/−^ worms (Fig. 2, D and E, and fig. S5, B and C). This indicates that restoring functionality in the Golgi alone is insufficient to rescue *lci-1* phenotypes and that the lysosomal fraction of human LCI is physiologically relevant.

To understand why human LCI rescues lysosome dysfunction in *lci-1* mutants, we measured pH and Ca^2+^ levels at single-lysosome resolution, using a previously described DNA-based, pH-correctable Ca^2+^ reporter, *CalipHluor2.0* (fig. S6, A and B, and table S2) (*17*, *27*). While lysosomal pH levels in *lci-1*^+/−^ worms did not vary from those in N2 worms, lysosomal Ca^2+^ levels are ~100-fold lower (Fig. 2, F and G, and text S2). Extrachromosomal expression of either WT human LCI or the R126C human LCI fully restores lysosomal Ca^2+^ levels, while G304R human LCI partially restores it (Fig. 2G and fig. S6, C and D). These results indicate that the heterologous expression of human LCI facilitates Ca^2+^ accumulation in nematode lysosomes, thereby restoring lysosome function and rescuing lethality.

### Mutations of putative Ca^2+^-binding sites impair human LCI activity

**Fig. 3.**
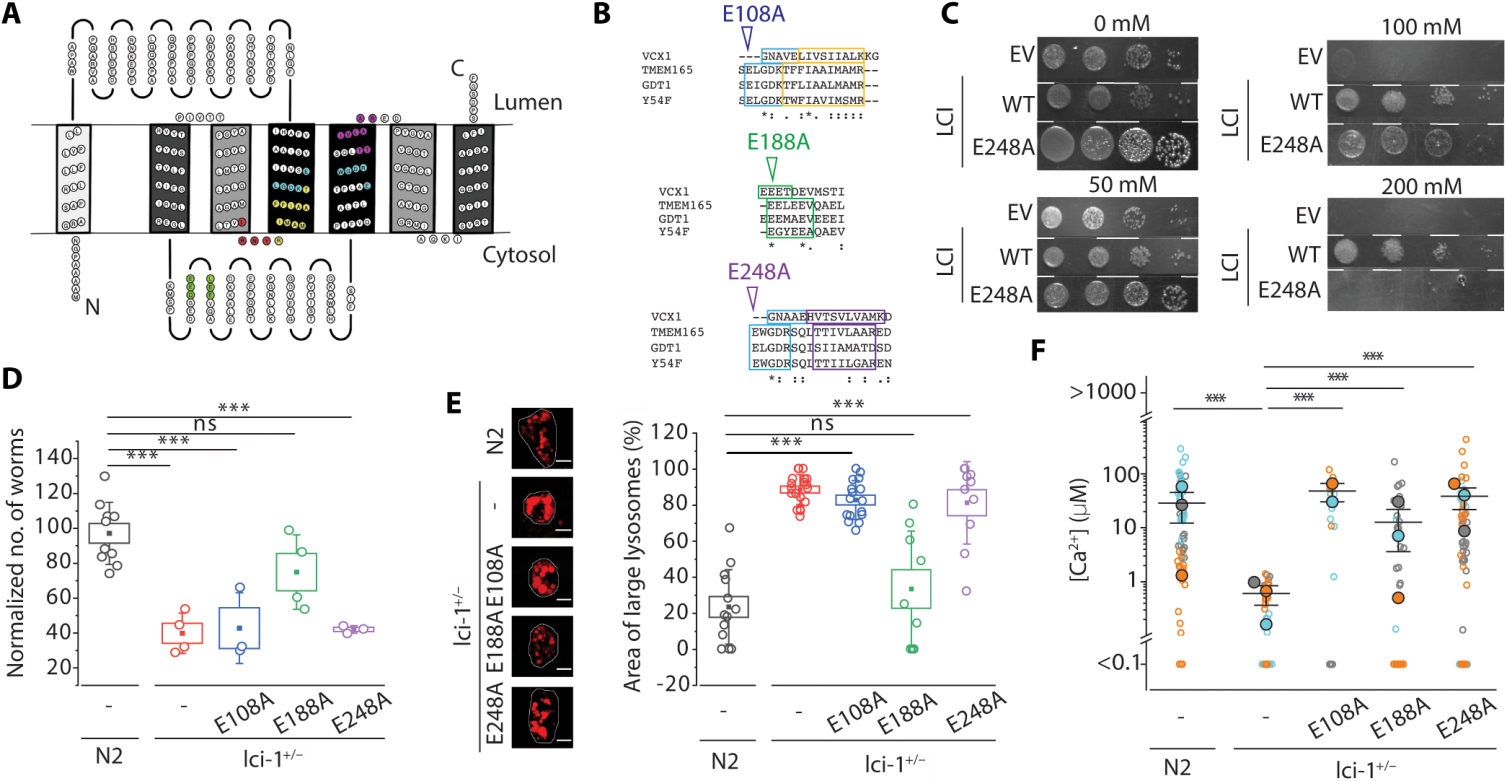
Homology with Vcx1 reveals potential calcium-binding sites in human LCI. (**A**) Topology of human LCI created using TOPO2. Grayscale shows the internal symmetry of transmembrane segments. Ca^2+^-binding regions (light blue), homologous regions adjoining the Ca^2+^-binding regions (yellow and purple), acidic helices (green), and lysosome/vacuole-targeting motifs (red) are highlighted. (**B**) Alignment of the Ca^2+^-binding sites (top and bottom) and acidic helix (middle) of Vcx1 (*S. cerevisiae*), TMEM165 (*H. sapiens*), *Y54F10AL.1* (*C. elegans*), and Gdt1 (*S. cerevisiae*) using Clustal Omega. Asterisks, colons, and periods indicate full, strong, and weak conservations, respectively. (**C**) Growth of K665 transformants integrating either an EV or the indicated variant of human LCI, after 2 days at 30°C on yeast extract, peptone, and dextrose plates supplemented with the indicated concentration of CaCl_2_. Columns indicate 10-fold dilutions from left to right. (**D**) Number of the progeny of *lci-1^+/−^* worms extrachromosomally expressing the indicated human LCI variant (*n* = 3 biological replicates). (**E**) Left: Representative fluorescence images of lysosomes in coelomocytes in worms of the indicated genetic background. Lysosomes are labeled with Alexa Fluor 647 duplex DNA. Right: Percentage of area occupied by enlarged lysosomes in the indicated genetic background. *N* > 5 cells, >50 lysosomes. (**F**) Distribution of lysosomal Ca^2+^ in the indicated genetic backgrounds. Data represent the means (closed blue, orange, and gray circles) of three independent experiments (open blue, orange, and gray circles). Lysosomes with Ca^2+^ levels below O/R_min_ or above O/R_max_ of *CalipHluor2.0* are plotted as <0.1 and >1000 μM, respectively. Scale bars, 5 μm. Boxes and bars represent the SEM and SD, respectively. ns, *P* > 0.05; *P < 0.05; **P < 0.01; ***P < 0.001 (one-way ANOVA with Tukey post hoc test).

Since human LCI was previously assigned as a Ca^2+^/H^+^ exchanger (CAX) (*21*, *28*), we tested whether it compensates the well-known *Saccharomyces cerevisiae* CAX protein, Vcx1, which imports excess cytosolic Ca^2+^ into the vacuole in exchange for vacuolar H^+^ (*29*, *30*). Despite its lack of homology with CAX families or other cation/Ca^2+^ (CaCA) transporters, we looked for shared structural features between human LCI and Vcx1. Human LCI and its orthologs belong to the UPF0016 family of membrane proteins of unknown function. UPF0016 family proteins contain two EXGDK/R motifs flanked by two hydrophobic regions (Fig. 3, A and B) (*23*). The acidic residues around the EXGDK/R motifs are posited to be involved in cation recognition (*24*) and previous studies suggest that human LCI conducts Ca^2+^ current (*21*, *31*, *32*). The structure of Vcx1 revealed a cytosolic loop rich in acidic amino acids that coordinates Ca^2+^ ions which are transported into the vacuole via a conformational change induced by the transmembrane (TM) H^+^ gradient (*29*). UPF0016 family proteins show twofold antiparallel symmetry, just like Vcx1 and other CaCA transporters (tables S3 and S4) (*21*, *33*). Human LCI has only seven predicted TM domains, unlike canonical CAX transporters that have ~14. However, it has regions with high homology to the acidic loop and to the helices in Vcx1 that bind Ca^2+^ (fig. S7A). These regions are highly conserved within the UPF0016 family and are adjacent to the EXGDK/R motif (Fig. 3B and fig. S7B). Furthermore, a homozygous missense E108G mutation in the EXGDK/R motif in TMEM165 leads to CDG type II in humans (*34*). A homology model of human LCI based on Vcx1 and other TM proteins shows the putative cation-binding region lining a pore with direct access to the acidic cytosolic loop, with the lysosome-targeting YNRL motif on the same side as the acidic helix (text S3, fig. S7C, and table S5). On the basis of these similarities, we tested whether human LCI activity is affected by mutating the putative cation-binding regions and in the acidic loop.

We exogenously expressed human LCI in an *S. cerevisiae* strain, denoted K665, that lacks both the vacuolar Ca^2+^ importers, *pmc1* and *vcx1* (*35*). High extracellular Ca^2+^ is toxic to K665 and exogenously expressing *Arabidopsis thaliana* CAX genes reverses this lethality (*35*). Exogenously expressing WT human LCI in K665 reverses its Ca^2+^ sensitivity (Fig. 3C, text S4, and fig. S8), while an E248A mutation in a putative cation-binding site of human LCI could not (Fig. 3C). Extrachromosomal expression of E108A or E248A human LCI variants, with mutations in either putative cation-binding site of human LCI, also failed to rescue the brood size and lysosome size defects of *lci-1*^+/−^ worms (Fig. 3, D and E, and fig. S9). This indicates that these mutations likely inactivate human LCI. An E188A mutation in the acidic loop led to partial LCI activity in these assays (Fig. 3, D and E, and fig. S9). None of these point mutations alter protein expression levels or lysosomal localization (fig. S10) indicating that the phenotypic differences are due to protein activity. Expression of all mutants in worms only partially rescued lysosomal Ca^2+^ levels (Fig. 3F, text 2, and fig. S11). Cumulatively, the results indicate that the EXGDK/R motif is vital for human LCI’s ability to elevate lysosomal Ca^2+^, as seen previously for Vcx1 (*31*). This suggests functional similarity between human LCI and Vcx1, despite their low overall structural homology.

### Human LCI facilitates Ca^2+^ entry into human lysosomes

We then tested whether human LCI facilitated Ca^2+^ entry into the lysosomes of human cells. First, we elevated cytosolic Ca^2+^ with adenosine 5′-triphosphate (ATP) in HeLa cells expressing human LCI, and tracked the cytosolic Ca^2+^ spike and its subsequent decrease by following Fura Red fluorescence with time (fig. S12A) (*36*). We also mapped lysosomal pH under identical conditions by imaging fluorescein isothiocyanate (FITC)–dextran–labeled lysosomes in whole cells (fig. S12A). An overlay of both traces reveals that lysosomal pH increases during the decay period of the cytosolic Ca^2+^ spike. Our findings are consistent with those of others, which led to them positing the existence of a pH-dependent lysosomal Ca^2+^ entry mechanism, such as Ca^2+^/H^+^ exchange (*14*, *16*). We therefore measured Ca^2+^ and pH in single lysosomes of HeLa cells where human LCI is either knocked out or overexpressed (fig. S12, B and C), using a *CalipHluor* variant called *CalipHluor^mLy^*, suited to probing mammalian lysosomes (fig. S12, D and E) (*17*). We found that, on average, human LCI deletion decreases lysosomal pH by ~0.5 U and decreases lysosomal Ca^2+^ by fivefold, which suggests a Ca^2+^/H^+^ exchange model of human LCI (fig. S13).

**Fig. 4.**
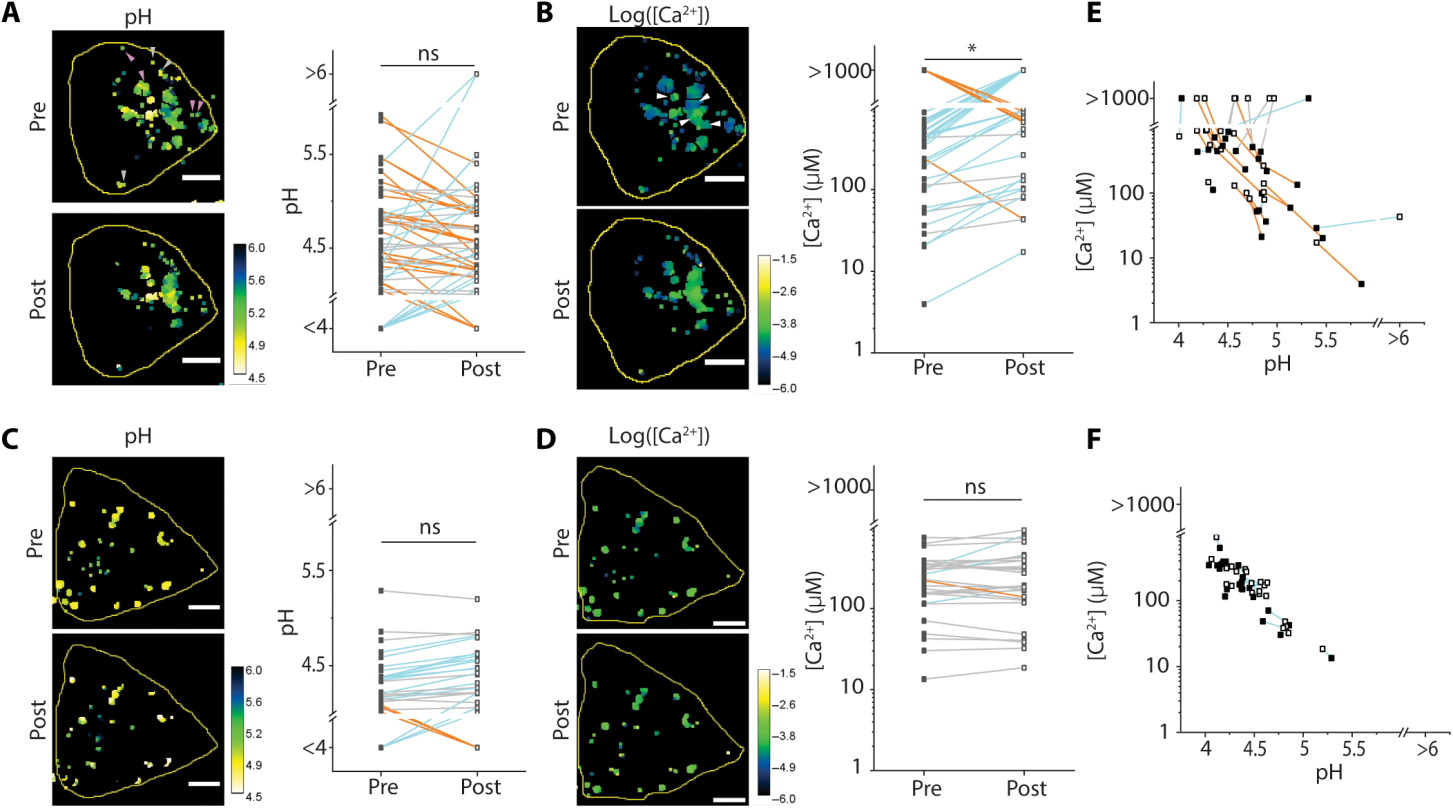
Ca^2+^ import into lysosomes by human LCI is associated with a lysosomal pH decrease. (**A**) Left: Representative pH maps of lysosomes from WT HeLa cells using *CalipHluor^mLy^* before and 2 min after 100 μM ATP addition. Arrowheads indicate lysosomes where pH decreases. Right: pH change of individual lysosomes of WT HeLa cells following the addition of ATP. Increasing, decreasing, and unchanged pH are indicated by blue, orange, and gray lines, respectively. (**B**) Left: Representative log([Ca^2+^]) maps of lysosomes from WT HeLa cells using *CalipHluor^mLy^* before and 2 min after 100 μM ATP addition. Arrowheads indicate lysosomes where [Ca^2+^] increases. Right: [Ca^2+^] changes in individual lysosomes of WT HeLa cells following ATP addition. Increasing, decreasing, and unchanged [Ca^2+^] are indicated by blue, orange, and gray lines, respectively. (**C**) Representative pH maps of lysosomes from *TMEM165* KO HeLa cells and pH change of individual lysosomes from *TMEM165* KO HeLa cells, as described in (A). (**D**) Representative log([Ca^2+^]) maps of lysosomes from *TMEM165* KO HeLa cells and [Ca^2+^] changes in individual lysosomes of *TMEM165* KO cells, as described in (B). (**E**) 2-IM maps of pH and [Ca^2+^] changes in WT HeLa cells before (closed square) and after (open square) adding ATP. Each data point indicates a single lysosome. Orange lines indicate lysosomes whose pH decreases and Ca^2+^ increases, cyan lines indicate lysosomes whose pH increases and Ca^2+^ increases, and gray lines indicate lysosomes whose Ca^2+^ decreases. (**F**) 2-IM maps of pH and [Ca^2+^] changes in *TMEM165* KO HeLa cells before (closed square) and after (open square) adding ATP, as described in part (E). Scale bars, 5 μm. ns, *P* > 0.05; *P < 0.05 (one-way ANOVA with Tukey post hoc test).

To directly visualize Ca^2+^/H^+^ exchange in single lysosomes due to human LCI activity, we simultaneously measured the pH and Ca^2+^ of single lysosomes at two different times (fig. S12A). One time point was in single lysosomes before cytosolic Ca^2+^ elevation. The other was 2 min after ATP addition corresponding to the Ca^2+^ spike, when the lysosomal Ca^2+^ entry mechanism, if any, is expected to be active. Our findings enforced a reconsideration of the Ca^2+^/H^+^ exchange model. We observed that after the cytosolic Ca^2+^ spike, pH increased only in 20% of lysosomes, decreased in 50% of lysosomes, and was unchanged in the remaining 30% (Fig. 4A), revealing the limitations of population-averaged lysosome measurements. Yet, Ca^2+^ increases in about 72% of lysosomes (Fig. 4B). When we performed the same experiments in *TMEM165* KO HeLa cells, the population of lysosomes where pH decreased in response to high cytosolic Ca^2^ was lost (Fig. 4, C and D).

To better reveal the missing lysosome population when human *LCI* was knocked out, we plot the pH of every lysosome versus its Ca^2+^ level to give a scatter plot, called a two-ion measurement plot, or 2-IM plot. 2-IM plots have been previously used to resolve lysosome populations in live cells (*37*, *38*). The 2-IM plots of HeLa cells expressing (Fig. 4E) and lacking human LCI (Fig. 4F) show lines connecting the same lysosome pre- and post-ATP addition. For clarity, orange lines show those lysosomes where pH decreases but Ca^2+^ increases, while cyan lines show lysosomes where both pH and Ca^2+^ increase, post-ATP addition. Note that Ca^2+^/H^+^ exchange is expected to favor the cyan population. Yet, after the cytosolic Ca^2+^ spike, nearly 75% of lysosomes where Ca^2+^ increased, clearly became more acidic. This is the opposite of what is expected if TMEM165 was a CAX as previously posited.

There are two possible explanations for the concurrent increase in Ca^2+^ and H^+^ levels in single lysosomes. One is an active mechanism such as pH-dependent Ca^2+^ transport (*14*, *39*). Alternatively, lumenal Ca^2+^ could get passively elevated because higher acidity protonates Ca^2+^-binding lysosomal proteins, making them release their bound Ca^2+^. The contribution of the passive increase in free Ca^2+^ is reflected in the negative slope of both 2-IM plots—pre- and post-Ca^2+^ spike—in TMEM165 KO cells (fig. S14, A and B). When human LCI is absent, the contribution of the passive mechanism is similar for both low and high cytosolic Ca^2+^ since protein content in single lysosomes is not expected to differ between both time points. The passive mechanism is also evident in HeLa cells expressing human LCI, but only at low cytosolic Ca^2+^ (fig. S14C). However, at high cytosolic Ca^2+^, the slope increases, indicating a much stronger coupling between H^+^ and Ca^2+^ levels in lysosomes when human LCI is present (fig. S14D).

This reveals that human LCI uses a mechanism whereby Ca^2+^ import becomes more efficient with higher lysosomal acidity. To test this dependence more explicitly, we evaluated the ability of human LCI to mop up cytosolic Ca^2+^ when the pH gradient across the lysosomal membrane is abolished. Inhibiting v-ATPase with bafilomycin A prevented lysosomal-localized human LCI from effectively restoring cytosolic Ca^2+^ to low, resting cell levels (fig. S15, A to D). We then sought to compare these findings in a cell line with low endogenous expression of human LCI, such as COS-7 cells (*40*). In these cells, low levels of human LCI lead to a much slower restoration of low cytosolic Ca^2+^ after a cytosolic Ca^2+^ spike (fig. S15, E to G). Overexpressing either WT human LCI or its lysosome-favoring mutant (R126C) speeds up the restoration of low cytosolic Ca^2+^, but not when the pH gradient across the lysosomal membrane is collapsed (fig. S15, E to G). Thus, the import of Ca^2+^ into lysosomes by human LCI requires an acidic lysosomal lumen.

### Electrophysiological characterization of human LCI

**Fig. 5.**
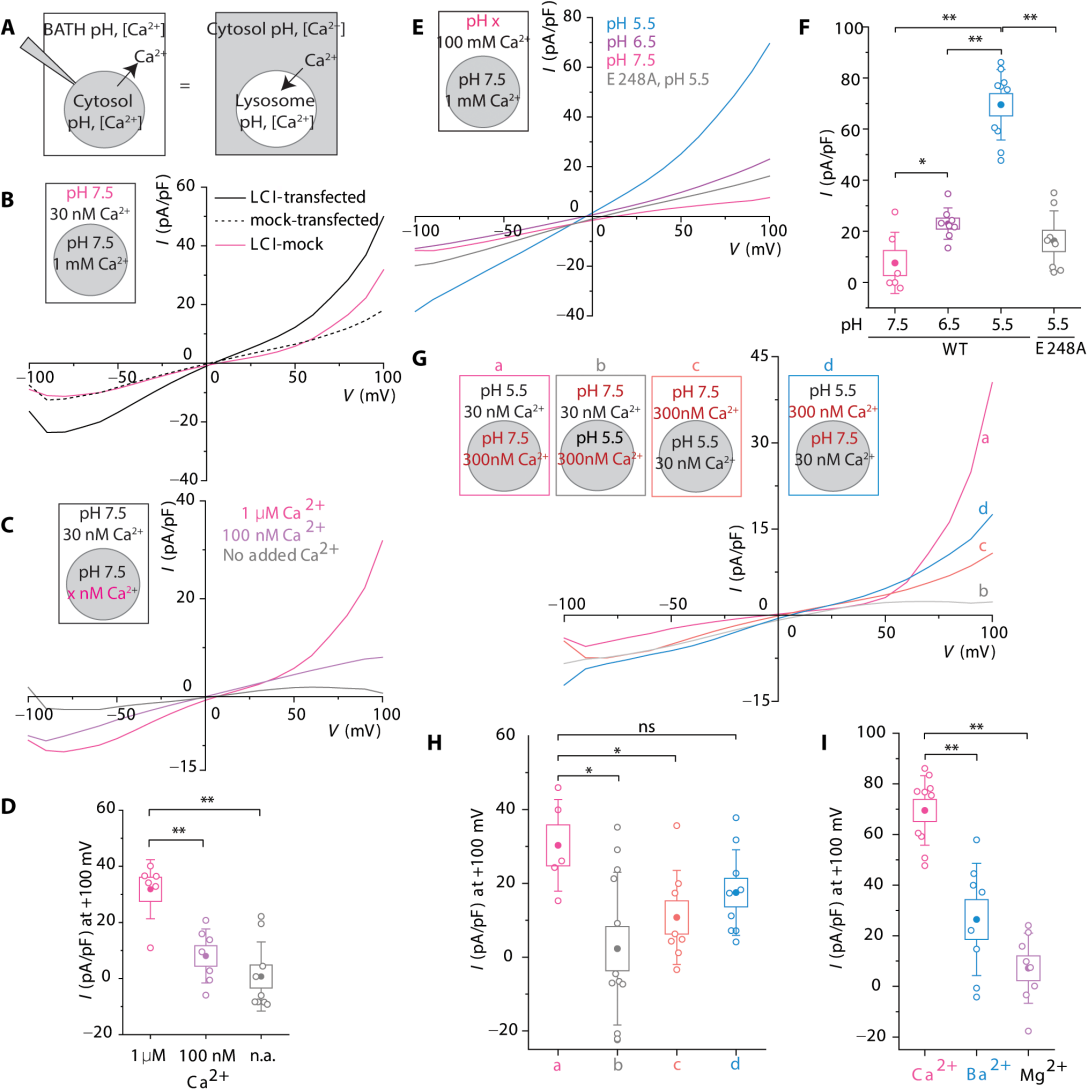
Human LCI conducts a unidirectional pH gradient-sensitive Ca^2+^ current. (**A**) Schematic of whole-cell electrophysiology used to record human LCI currents. The bath/extracellular solution is equivalent to the lysosomal lumen, and the pipette solution is equivalent to the cytosol. Outward (positive) current represents Ca^2+^ transport from the cytosol to the bath/lysosome lumen. (**B**) Average current densities of mock-transfected HeLa cells (dashed line), human *LCI*-transfected HeLa cells (solid black line), and mock-subtracted human LCI current density (solid magenta line). (**C**) Average current densities of mock-subtracted human LCI current in HeLa cells under the indicated conditions, with no added Ca^2+^ (gray), 100 nM Ca^2+^ (mauve), or 1 μM Ca^2+^ (magenta). (**D**) Current density at +100 mV for the indicated conditions in (C). (**E**) Average current density of mock-subtracted E248A (gray) or WT human LCI current in HeLa cells under the indicated conditions, with an extracellular pH of 7.5 (magenta), 6.5 (mauve), or 5.5 (blue). (**F**) Current density at +100 mV for the indicated conditions in (E). (**G**) Average current density of mock-subtracted human LCI current in HeLa cells for the indicated conditions, with either 30 or 300 nM Ca^2+^ and pH 5.5 or 7.5 in the cytosol and bath. (**H**) Current densities at +100 mV for the indicated cell conditions from (G). (**I**) Average current density at +100 mV for conditions indicated in fig. S16F. Boxes and bars represent the SEM and SD, respectively. ns, *P* > 0.05; *P < 0.05; **P < 0.01; ***P < 0.001 (one-way ANOVA with Tukey post hoc test).

To test whether human LCI directly transported Ca^2+^ across cellular membranes, we performed whole-cell patch-clamp electrophysiology using *N*-methyl-d-glucamine (NMDG)–methane sulfonic acid (MSA) buffers, where Ca^2+^ was the only transportable ion. As human LCI overexpression leads to adequate cell surface expression (fig. S16A), we mimicked, at the plasma membrane, human LCI activity across the lysosome membrane by using NMDG-MSA bath and pipette buffers that mimicked lysosomal and cytosolic ionic environments, respectively (Fig. 5A and fig. S16B) (*28*). In the absence of a TM pH gradient, HeLa cells overexpressing human LCI showed higher outward currents than mock-transfected HeLa cells indicating the movement of Ca^2+^ from the cytosol into the bath/lysosome even at low cytosolic Ca^2+^ (Fig. 5B and fig. S16C). To explicitly test whether human LCI transports Ca^2+^, we progressively reduced Ca^2+^ in the cytosol/pipette buffer and recorded currents as a function of the Ca^2+^ gradient alone (Fig. 5C). With only an inward Ca^2+^ gradient, we observed a substantial current density of 30 pA/pF at 100 mV with the direction of current matching the flow of Ca^2+^ down the gradient. When cytosolic Ca^2+^ is reduced to trace levels, where pipette/cytosolic buffer contains only EGTA, two things change: the outward current decreases threefold, and the reversal potential shifts toward slightly more positive values (Fig. 5, C and D, and fig. S16D). Thus, human LCI directly transports Ca^2+^ across cellular membranes.

We then studied the pH dependence of Ca^2+^ transport by human LCI. We recorded currents under increasing TM pH gradients, by increasing the acidity of the bath/lysosomal buffer while keeping the inward Ca^2+^ gradient constant (Fig. 5E). We found that the outward currents conducted by human LCI increased with increasing acidity of the bath/lysosomal buffer, after subtracting mock-transfected currents under identical conditions (Fig. 5, E and F). Thus, the transport of Ca^2+^ by human LCI increases with increasing acidity of the bath/lysosomal buffer. Overexpressing human LCI with an alanine mutation in its putative cation-binding site (E248A) reduced the outward current nearly three- to fourfold, even at high TM pH gradients, indicating that this mutation heavily compromises Ca^2+^ transport (Fig. 5, E and F).

TMEM165 was previously postulated to be a CAX because it feebly resembled the vacuolar CAX, Vcx1. However, electrophysiology as a function of pH reveals reversal potential shifts of <10 mV in the negative direction for a 100-fold difference in TM H^+^ (∆pH) which is miniscule for a CAX (fig. S17A). Even a 10-fold change in [∆H^+^] should shift the reversal potential by ~50 to 180 mV in the positive direction for reasonable stoichiometries (table S6). Thus, although protons enhance Ca^2+^ transport, H^+^ transport per se by human LCI is meager (Fig. 5E).

Exchangers are able to switch their ion transport directionality depending on the TM ion gradient. Therefore, we tested the direction of Ca^2+^ transport by human LCI at high (300 nM) or low (30 nM) cytosolic Ca^2+^; conditions expected to mimic the direction of Ca^2+^ flow across the lysosomal membrane in stimulated or resting cells, respectively (Fig. 5G, a and d). Regardless of the direction of the TM Ca^2+^ gradient, human LCI conducts predominantly outward Ca^2+^ current (Fig. 5, G and H). Since lysosome lumens are normally acidic, these results indicate that human LCI imports Ca^2+^ into lysosomes regardless of the degree or direction of the lysosomal TM Ca^2+^ gradient. To try and reverse the direction of Ca^2+^ transport as is expected of an exchanger, we reversed the TM pH and Ca^2+^ gradients and recorded currents (Fig. 5G, b and c). At acidic cytosolic pH, outward currents reduced ~4-fold, while inward current changes were negligible (Fig. 5H and fig. S17B). Thus, human LCI is only capable of transporting Ca^2+^ unidirectionally into the lysosome and is pH-activated only at the extracellular/lumenal face of the membrane. Further, the current amplitude through human LCI as a function of divalent cations varies as Ca^2+^ > Ba^2+^ ~ Mg^2+^ (Fig. 5I and fig. S17C). This behavior is unlike that of Ca^2+^ channels and more consistent with that of Ca^2+^ uniporters, such as MCU, that show a strong preference for Ca^2+^ (*41*–*43*). The comparison between MCU and LCI does not extend to the Ca^2+^-dependent change in reversal potential, which appears smaller or unchanged for LCI, compared to MCU (*44*). The higher rectification of MCU currents makes this comparison complicated, and further studies are needed to address this discrepancy in the context of the differences in their Ca^2+^ import mechanisms.

Last, we directly interrogated human LCI current on isolated swollen lysosomes of COS-7 cells, whose flatter morphology lends itself to lysosome patching. Both WT and R126C human LCI-EGFP can be visualized on lysosomes of COS-7 cells upon treatment with vacuolin-1, which swells lysosomes (fig. S18, A and B) (*45*). We used pipette buffers to mimic the lumenal acidity of either lysosomes or the Golgi (fig. S18C). Here too, lysosomes of cells expressing human LCI show outward current (positive current into the lysosome) under either condition. At +100 mV, the current at lysosomal pH was ~2.5-fold higher than at pH levels corresponding to the Golgi (fig. S18, D and E). Cumulatively, our data are consistent with human LCI being a proton-activated, Ca^2+^ importer in lysosomes.

## DISCUSSION

The high abundance of TMEM165 on the Golgi has led to detailed studies into its roles in protein glycosylation and its effects on human physiology (*22*, *32*, *46*–*48*). While the most common pathophysiology of TMEM165 is linked to Golgi dysfunction, its significance in lysosomes has thus far been overlooked. For example, in a patient with heterozygous missense mutations (c.376C>T [p.R126C]) and (c.910G>A [p.G304R]), human LCI is absent in lysosomes. Despite its presence on the Golgi, this patient manifested a less severe form of CDG-II, presenting with characteristic deformities that are also observed in lysosomal storage disorders such as fucosidosis and Gaucher’s disease (*24*).

While human LCI performs important Golgi-related roles, our data now show that it has an additional function, namely, importing Ca^2+^ into lysosomes. Human LCI was previously posited to act like a CAX at the Golgi, based on its weak structural homology with Vcx1. However, our studies reveal that human LCI imports Ca^2+^ into organelle lumens by acting as a proton-activated Ca^2+^ importer. Human LCI is roughly half the size of a canonical CAX and could therefore potentially import Ca^2+^ as a homo or heterodimer. We therefore suggest a model of human LCI as a “broken CAX” whose Ca^2+^ transport function is preserved, but where it cannot transport the protons that trigger its activity.

Lysosomal Ca^2+^ import is a key function in cells, and, while the existence of a pH-dependent Ca^2+^ import mechanism has long been known, the identity of the gene responsible was not. We now identify human LCI as a pH-activated lysosomal Ca^2+^ importer in humans. Mutations in every known lysosomal Ca^2+^ channel disrupt lysosomal Ca^2+^ homeostasis and lead to diverse neurological disorders. Given that human LCI can restore lysosomal Ca^2+^ channel phenotypes, it could provide previously unidentified avenues to explore disorders arising from defective lysosomal Ca^2+^ channels.

## MATERIALS AND METHODS

### Chemicals and reagents

Modified oligonucleotides (table S2) were purchased from IDT (USA), subjected to ethanol precipitation, and quantified using ultraviolet absorbance. Chemicals used for the previously synthesized Rhod-5F-azide were purchased from Sigma-Aldrich and Alfa Aesar as previously described (*17*). Dichlorofluorescein (DCF) used for previously described conjugation was purchased from Thermo Fisher Scientific (*49*). Bafilomycin A and vacuolin-1 were purchased from Cayman Chemical. EGTA, ampicillin, kanamycin, carbenicillin, ammonium chloride, adenosine triphosphate, yeast synthetic dropout media, Triton X-100, bovine serum albumin (BSA), glyoxal, MSA, Hepes, calcium hydroxide, magnesium chloride, barium hydroxide, and NMDG were purchased from Sigma-Aldrich. Monodisperse silica microspheres were obtained from Cospheric. TMR-dextran (10 kDa), Fura Red, Alexa Fluor 488 NHS Ester, and ribonuclease (RNase) A were purchased from Thermo Fisher Scientific. Yeast Nitrogen Base (YNB) with ammonium sulfate was purchased from MPBio. Rabbit anti-TMEM165 was purchased from ProteinTech. Mouse anti-GM130 was purchased from Santa Cruz Biotechnology. Alexa Fluor 488 Goat anti-Rabbit, Alexa Fluor 647 Goat anti-Mouse, and GFP Polyclonal Antibody Alexa Fluor 555 were purchased from Thermo Fisher Scientific. Paraformaldehyde (PFA) and glycine were purchased from Thermo Fisher Scientific. Zymolyase was purchased from Zymo Research. Anti-pan cadherin antibody was purchased from Abcam.

### Mammalian cell culture, plasmids, and transfection

HeLa cells and COS-7 cells were purchased from the American Type Culture Collection and cultured according to recommended guidelines. *TMEM165* KO HeLa cells were purchased from Creative Biogene. Cells were cultured in Dulbecco’s modified Eagle’s medium (DMEM) (Invitrogen Corporation, USA) containing 10% heat-inactivated fetal bovine serum (Invitrogen Corporation, USA), penicillin (100 U/ml), and streptomycin (100 μg/ml) (Gibco) and maintained at 37°C under 5% CO_2_. Cells were passaged using 0.25% Trypsin-EDTA (Gibco) and plated at 50 to 60% confluency for transfection.

For mammalian expression of human LCI fused to EGFP, the cDNA of *TMEM165* (Harvard Medical School Plasmid Database) was cloned into the pEGFP-N1 plasmid, which was obtained from M. Fransen (KU Leuven). Cloning was done using Gibson assembly techniques. Briefly, inserts and backbones were amplified using polymerase chain reaction (PCR), using LongAmp Taq DNA Polymerase (NEB) and primers with 5′ overhangs enabling 15 to 20–base pair (bp) overlap of the insert and backbone. Amplification was verified on 0.8% agarose gel before digestion of starting templates with DpnI (NEB) and purification of PCR products (DNA Clean and Concentrator, Zymo Research). At least a twofold molar excess of the insert was added to the backbone for the Gibson reaction using the Gibson Assembly Master Mix (NEB). The assembly reaction was transformed into competent DH5α *Escherichia coli* cells, and colonies grown in kanamycin were inoculated in liquid LB with ampicillin. The plasmid DNA was isolated via minipreparation (GeneJET Plasmid Miniprep Kit, Thermo Fisher Scientific) and verified by sequencing, using forward and reverse primers upstream and downstream of the ligation sites. For mammalian expression of human LCI fused to DsRed, the DNA for DsRed (a gift from B. Dickinson at the University of Chicago) was cloned into the human LCI-EGFP plasmid, replacing EGFP, using Gibson assembly techniques.

For mammalian expression of human LCI disease mutants fused to EGFP, the above construct was subject to site-directed mutagenesis using the Q5 Site-Directed Mutagenesis Kit (NEB). The R126C mutant was generated using forward primer GCGCTATAACTGCCTGACCGT and reverse primer ATTGCCATGATGGCTGCTATAAAAAATG. The R126H mutant was generated using the forward primer CGCTATAACCACCTGACCGTG and the reverse primer CATTGCCATGATGGCTGC. The G304R mutant was generated using the forward primer GACAATCATAAGAGGCATCGTTTTTTTG and the reverse primer ACAGTTCTGACAGAGATTTTC. Resulting plasmids were verified by sequencing. The GFP-Rab7 plasmid was a gift from R. Pagano (Addgene, plasmid #12605).

HeLa and COS-7 cells were transiently transfected with the respective plasmids using Lipofectamine 3000 (Thermo Fisher Scientific) according to the manufacturer’s protocols. After incubation for 4 hours, the transfection medium was replaced with fresh DMEM. Imaging or electrophysiology experiments were performed on cells 48 hours following transfection.

### *C. elegans* methods and strains

Standard methods were used to maintain *C. elegans* (*50*). The WT strain was the *C. elegans* isolate from Bristol, strain N2. Three strains used in the study were provided by the Caenorhabditis Genetics Center: *+/mT1 II; cup-5(ok1698)/mT1 [dpy-10(e128)] III*, a heterozygous KO of *cup-5* balanced by *dpy-10*–marked translocation; *arIs37[myo-3p::ssGFP + dpy-20(+)]I*, a transgenics strain expressing soluble GFP (ssGFP) in the body muscles which is secreted into the pseudocoelom and endocytosed by coelomocytes, and *arIs37[myo-3p::ssGFP + dpy-20(+)]Icup5(ar465)*, a transgenic strain with enlarged GFP-containing vesicles in coelomocytes due to a point mutation in *cup-5*. The strain with a heterozygous KO of *lci-1* was generated and provided by D. Moerman (University of British Columbia) using CRISPR-Cas9 technology and verified by sequencing (*51*). Heterozygous *lci-1*^+/−^ worms are marked by pharyngeal GFP, homozygous *lci-1*^+/+^ progeny is functionally WT but lacks GFP, and homozygous *lci-1*^−/−^ progeny is embryonic lethal. The genotype of this worm is *Y54F10AL.1(gk5484[loxP + Pmyo-2::GFP::unc-54 3′UTR + Prps-27::neoR::unc-54 3′UTR + loxP])/+ III*. Transgenic strains expressing human LCI variants were generated by microinjecting plasmid DNA into *lci-1^+/−^* gonads to produce extrachromosomal arrays. The injected plasmid contained a human LCI variant with the promoter region of *lci-1* and the 3′ untranslated region (3′UTR) of *unc-54*. Pharyngeal mCherry was used as an injection marker. The plasmid construction, worm injection, and verification by sequencing were performed by SunyBiotech (Fujian, China) using established protocols.

Gene knockdown was performed using Ahringer Library–based RNAi methods (*52*). The RNAi clones used were L4440 empty vector (EV) control, *catp-6* (W08D2.5, Ahringer Library), *cup-5* (R13A5.1, Ahringer Library), *lci-1* (Y54F10AL.1, Ahringer Library), *ncx-1* (Y113G7A.4, Ahringer Library), *ncx-2* (C10G8.5, Ahringer Library), *ncx-3* (ZC168.1), *ncx-4* (F35C12.2), and *clh-6* (R07B7.1, Ahringer Library).

### Survival assay in *C. elegans*

The *N2*, *+/mT1 II; cup-5(ok1698)/mT1 [dpy-10(e128)] III*, and *Y54F10AL.1(gk5484[loxP + Pmyo-2::GFP::unc-54 3′UTR + Prps-27::neoR::unc-54 3′UTR + loxP])/+ III* nematode strains were used for this assay, which was performed as previously described (*17*).

To screen lysosomal genes for calcium import activity, hundreds of L1-L2 worms of the indicated genotype were placed on fresh plates with OP50 and allowed to grow to L4 for 2 days. Five of these L4 worms were placed on plates containing bacterial strains L4440 with RNAi for the indicated genes. The worms were allowed to grow for 24 hours and lay eggs, after which the adult worms were removed from the plates. Eggs were allowed to hatch and grow into adults for 3 days. The worm plates were scanned twice on an Epson Perfection v850 Scanner in batches of 12 plates. The average number of worms on the plates between the two scans was calculated using the Fiji plug-in “WormAnalysisProgram.” The difference in brood size and statistical significance were calculated with respect to the EV.

Where small-scale RNAi was performed, five L4 worms of the indicated strain were placed on plates containing the indicated RNAi bacterial strains. Where RNAi was not performed, five L4 worms of the indicated strain were placed on OP50 plates. The worms were allowed to grow for 24 hours and lay eggs, after which the adult worms were removed from the plates. Eggs were allowed to hatch and grow into adults for 3 days. The worm plates were imaged under an Olympus SZX-Zb12 Research Stereomicroscope (Olympus Corporation of the Americas) with a Zeiss Axiocam color CCD camera (Carl Zeiss Microscopy). Images were then analyzed using ImageJ to count the number of adult worms per plate.

### Lysosome size assay in *C. elegans*

The *N2*, *arIs37[myo-3p::ssGFP + dpy-20(+)]I*, *arIs37[myo-3p::ssGFP + dpy-20(+)]Icup5(ar465)*, and *Y54F10AL.1(gk5484[loxP + Pmyo-2::GFP::unc-54 3′UTR + Prps-27::neoR::unc-54 3′UTR + loxP])/+ III* nematode strains were used for this assay, which was performed as previously described (*17*). Briefly, five L4 worms were placed on either OP50 plates or plates containing the indicated RNAi bacterial strains. After 24 hours of laying eggs, the adult worms were removed from the plates. After 3 days, the adult worms are imaged to check for lysosome size differences. Worms were imaged on a confocal microscope for either ssGFP-labeled coelomocyte lysosomes or a DNA duplex with Alexa Fluor 647 (table S2). Lysosome areas were measured using ImageJ, and enlarged lysosomes were defined as those whose diameter is ≥33% of the diameter of the largest lysosomes in N2 worms. Lysosome size data were plotted as the percentage of area occupied by large lysosomes relative to the total lysosomal area.

### Survival assay in *S. cerevisiae*

The yeast strain K665 (*pmc::TRP1 vcx*Δ) was a gift from K. Hirschi (Baylor College of Medicine). The yeast integrating plasmid YIplac128 was a gift from B. Glick (University of Chicago). Cloning of human LCI into YIplac128 was done using Gibson assembly techniques, as described above. The K665 strain was transformed with either the YIplac128 EV or YIplac128 containing human LCI using Frozen-EZ Yeast Transformation II Kit (Zymo Research) according to the manufacturer’s protocols and transformed colonies were selected following growth on Synthetic Dropout Leucine (SD-Leu) agar plates overnight at 30°C. Colonies were then grown in SD-Leu liquid media while shaking at 30°C until cultures had an optical density (OD) of ~0.6. For the survival assay, a series of 10-fold dilutions of each culture was prepared and dropped onto SD-Leu agar plates with the indicated amounts of added CaCl_2_. After 48 hours of growth, plates were removed and visualized for growth.

### RNAi and RT-PCR

Bacteria from the Ahringer RNAi library (obtained as a gift from F. Ausubel, Massachusetts General Hospital) expressing double-stranded RNA against the relevant gene were fed to worms, and the relevant experiments were carried out in 1-day-old adults of the F1 progeny. RNA knockdown and genetic background were confirmed by probing messenger RNA levels of the candidate gene, assayed by RT-PCR. Briefly, total RNA was isolated using the TRIzol-chloroform method and 1 μg of total RNA was converted to complementary DNA using oligo-dT primers and SuperScriptIV RT according to the manufacturer’s protocols. Then, 5 μl of cDNA product was used to set up a PCR using gene-specific primers for *Y54F10AL.1* (F: ATTCCACCGATTTCCACCCC, R: CTTCTTGGCCCTCATTCGGT, 527 bp) *ncx-1* (F: ACAACTACAAATGCGATGACCA, R: ATTGTCGATGGTCCCAGCTC, 515 bp), *ncx-2* (F: TTCGCTACCATCCCACCAAC, R: CAGTAGGCTTCCAATGCGGA, 539 bp), *ncx-3* (F: CGGTTTGGTGACTGCTGTTG, R: CGTAGACAATCCAGAGGCCC, 449 bp), *ncx-4* (F: GTCTACCGATTCCGTGGCTT, R: CCGTTGATGCAGACCGTTTT, 359 bp), *clh-6* (F: ACGACTGCAGGGTGTATGTG, R: CGAGACCTGTCCATGAGAGC, 574 bp), *catp-6* (F: ATTGTTGGTGCAGTCAACGC, R: GGGGGAATGTTATGCAAGTCG, 503 bp), *cup-5* (F: GCGGTTAGAGCAAATTCCCC, R: TGAGCGCCAAGATTTCCAGA, 561 bp), TMEM165 (F: CACCAGCAGCTCCAGTTCAT, R: TGAGAGCGATCACCCCATTC, 544 bp), and *act-1* (F: TGCAGAAGGAAATCACCGCT, R: AGAAAGCTGGTGGTGACGAT, 251 bp) where indicated. PCR products were separated on a 0.8% agarose–tris base, acetic acid, and EDTA (TAE) gel.

To confirm the expression of human LCI in the *S. cerevisiae* strain K665, RNA levels were probed using RT-PCR. Genomic DNA (gDNA) was isolated using Monarch Genomic DNA Purification Kit (NEB) according to the manufacturer’s protocols. Briefly, transformants were grown overnight in liquid SD-Leu media, and cells were harvested by centrifugation for 1 min at 12,000*g*. Cells were resuspended in lysis buffer with 10 μl of zymolyase and 3 μl of RNase A and incubated for 30 min at 37°C. Then, 10 μl of proteinase K and 100 μl of tissue lysis buffer were added and the mixture was vortexed thoroughly before incubating for 30 min at 56°C with shaking. Then, 400 μl of gDNA binding buffer was added, and the mixture was vortexed before transferring to a purification column in a collection tube. The column was centrifuged for 3 min at 1000*g* and then 1 min at 12,000*g*. Then, the column was washed twice with 500 μl of gDNA wash buffer before adding 50 μl of pre-heated gDNA elution buffer. The column was incubated for 1 min and centrifuged into a microforge tube for 1 min at 12,000*g*. The purity and amount of DNA were determined using a NanoDrop, and 20 ng of DNA was used to run a PCR using gene-specific primers for TMEM165 (same primers as above) and ACT1 (F: GAAATGCAAACCGCTGCTCA, R: GAGCCAAAGCGGTGATTTCC, 289 bp). PCR products were separated on a 0.8% agarose-TAE gel.

### Localization in yeast

Cloning of human LCI-DsRed into YIplac128 was done using Gibson assembly techniques, as described above. The K665 strain was transformed with either the YIplac128 EV or YIplac128-LCI-DsRed as described above, and transformed colonies were selected following growth on SD-Leu agar plates overnight at 30°C. Colonies were then grown in SD-Leu liquid media while shaking at 30°C until cultures had an OD of ~0.6. Vacuoles were then labeled with the dye FM4-64, according to the manufacturer’s protocols. Briefly, yeast cultures were harvested by spinning down for 1 min at 5000*g* for 5 min. The supernatant was discarded and the cell pellet was resuspended in 50 μl of yeast extract, peptone, and dextrose (YPD) media and 1 μl of 1.6 μM FM4-64. This mixture was incubated for 20 min in a 30°C water bath. Then, 1 ml of YPD media was added and the mixture was centrifuged for 5 min at 5000*g*. The supernatant was discarded and the cells were resuspended in 5 ml of YPD media. This mixture was incubated in a shaker for 90 min at 30°C. The cells were centrifuged for 5 min at 5000*g* before removing the supernatant and resuspending cells in 1 ml of sterile water. Cells were then spun down again for 5 min at 5000*g* before resuspending in 25 μl of YPD media. On a coated glass slide, 7 μl of the sample was spotted and covered with a coverslip. Cells were then imaged on a widefield microscope (see the “Image acquisition” section below for details).

### Colocalization in live cells

To evaluate the subcellular localization of human LCI mutants, we analyzed colocalization with TMR-dextran or FITC-dextran in live cells. In both experiments, COS-7 cells were transfected with the indicated variant of human LCI-EGFP or human LCI-DsRed with Lipofectamine 3000 according to the manufacturer’s protocol and either imaged or fixed 48 hours later. In the live cell experiments, transfected COS-7 cells were pulsed with TMR-dextran (1 mg/ml) or FITC-dextran (5 mg/ml) for 1 hour and chased for 16 hours before imaging. Where indicated, COS-7 cells were also incubated in 5 μM vacuolin-1 for 24 hours before imaging on a confocal microscope (see the “Image acquisition” section for details). Pearson’s correlation coefficient (PCC) was calculated using software in ImageJ, before and after a pixel shift. No threshold PCC was used for colocalization with TMR-dextran or FITC-dextran.

### Immunofluorescence

In anti-GM130 experiments, COS-7 cells were transfected with the indicated variant of human LCI-EGFP. Two days later, they were washed three times with phosphate-buffered saline (PBS) and fixed using 4% paraformaldehyde at room temperature (RT) for 10 min. Then, cells were washed three times with PBS and permeabilized with 0.2% Triton X-100 for 5 min before washing again and blocking with 3% BSA for 60 min. Cells were then incubated with 1:1000 anti-GM130 and 1:1000 anti-TMEM165 in 0.3% BSA overnight at 4°C. Last, cells were washed three times with PBS and incubated in 1:1000 goat anti-mouse Alexa Fluor 647 and 1:1000 goat anti-rabbit Alexa Fluor 488 in 0.3% BSA for 60 min before imaging on a confocal microscope (details in the “Image acquisition” section). Above-threshold PCC was used for colocalization with GM130.

For immunofluorescence to check human LCI expression, HeLa cells or TMEM165 KO HeLa cells were fixed, permeabilized, and blocked as above. Cells were then incubated with 1:1000 anti-TMEM165 in 0.3% BSA in PBS overnight at 4°C. Cells were washed three times with PBS and incubated in 1:1000 goat anti-rabbit Alexa Fluor 488 in 0.3% BSA in PBS for 60 min before imaging on a confocal microscope (details in the “Image acquisition” section). The background-subtracted whole-cell intensity was used to evaluate expression levels.

For immunofluorescence in cells with swollen lysosomes, HeLa cells were pretreated with 5 μM vacuolin-1 before fixation. Two days later, cells were washed three times with PBS and fixed using 1% glyoxal solution in PBS for 5 min. Then, cells were washed three times with PBS and permeabilized with 0.2% Triton X-100 for 10 min. Then, cells were washed three times with PBS and blocked with 3% BSA for 1 hour. Cells were then incubated in 1:100 mouse anti-LAMP1 and/or 1:200 rabbit anti-TMEM165 in 0.3% BSA overnight at 4°C. Last, cells were washed three times with PBS and incubated in 1:1000 goat anti-rabbit Alexa Fluor 488 and/or 1:1000 goat anti-mouse Alexa Fluor 647 in 0.3% BSA for 1 hour before imaging on a confocal microscope (details in the “Image acquisition” section).

For surface-only immunostaining, HeLa cells were first transfected with human LCI-EGFP. Two days later, cells were fixed with 2% PFA for 10 min on ice and washed three times with ice-cold PBS. Cells were then blocked with 3% BSA with 0.3 M glycine in PBS for 30 min. Cells were then incubated in 1:100 anti-GFP Alexa Fluor 555 and 1:500 rabbit anti-pan cadherin in a blocking buffer overnight at 4°C. After washing three times with PBS, cells were incubated in 1:1000 goat anti-rabbit Alexa Fluor 647 in a blocking buffer for 1 hour before washing and imaging on a confocal microscope (details in the “Image acquisition” section).

### Lysosomal pH and Ca^2+^ imaging in worms

In vivo lysosomal pH and Ca^2+^ measurements were made using *CalipHluor2.0* according to the protocols and calibrations established previously (*17*, *49*). The methods used are summarized briefly below.

#### CalipHluor2.0 preparation

In *CalipHluor2.0*, the Ca^2+^ indicator, Rhod-5F (O), fluoresces upon binding Ca^2+^ (*17*). The *K*_d_ of Rhod-5F for Ca^2+^ binding is pH-dependent, which is why *CalipHluor2.0* incorporates a pH-sensing dye DCF (G) (*49*). For ratiometric quantification of Ca^2+^ and pH, we incorporate Alexa Fluor 647 (R) as a reference dye. Thus, the G/R ratio maps the pH at every pixel and is used to obtain pH-corrected values of Ca^2+^ based on the O/R ratio at the single-lysosome resolution. *CalipHluor2.0* was prepared according to the previously reported procedure (*49*). Oligonucleotides used to form *CalipHluor2.0* are listed in table S2.

#### In vitro pH and calcium calibration

The calibration curve used here to make pH measurements with the DCF/Alexa Fluor 647 (G/R) ratio from *CalipHluor2.0* was prepared in (*38*). The resulting curve was fitted to Eq. 1

$$\text{pH}=pH_{1/2}+0.3\text{ln}\left(\frac{K_{1}-K_{2}}{Y-K_{2}}-1\right)$$ (1)

where *K*_1_, *K*_2_, and pH_1/2_ represent parameters from a Boltzmann fit of the calibration curve and *Y* represents the G/R ratio. An in vitro bead calibration of *CalipHluor2.0* at pH 5 on the same day of measurements (see below) was used to correct for day-to-day variation in the calibration curve. The *K*_d_ curve used to correct the effect of pH on Rhod-5F binding to Ca^2+^ was determined in (*11*). The curve was fitted to Eq. 2

$$K_{d}=1.03+\left(5.14*10^{12}*e^{-\frac{\text{pH}}{0.189}}\right)+\left(3.108*10^{6}*e^{-\frac{\text{pH}}{0.412}}\right)$$ (2)

To calculate the effect of pH on the fold change in the Rhod-5F/Alexa Fluor 647 (O/R) ratio of *CalipHluor2.0* from 0.1 μM to 1 mM free Ca^2+^, we used the fold-change calibration curve prepared in (*11*). The curve was fitted to Eq. 3 to get the minimum O/R (O/R at 0.1 μM Ca^2+^) as a function of pH and normalized to the maximum O/R (O/R at 1 mM Ca^2+^)

$$O/R_{\text{min}}=\frac{1}{4.24+0.12*e^{0.5*\text{pH}}}$$ (3)

An in vitro bead calibration of *CalipHluor2.0* was performed on the same day as measurements were made to correct for day-to-day variation in fold change, as previously described (*17*, *49*). Briefly, 500 nM of *CalipHluor2.0* was incubated with 0.6-μm monodisperse silica beads in 20 mM sodium phosphate buffer (pH 5.1) containing 500 mM NaCl for 30 min at RT. The beads were washed three times by spinning at 10,000 rpm for 10 min at RT. Beads absorbed with *CalipHluor2.0* were incubated with clamping buffer [Hepes (10 mM), MES (10 mM), sodium acetate (10 mM), EGTA (10 mM), KCl (140 mM), NaCl (500 mM), and MgCl_2_ (1 mM)] for 30 min at RT containing 0.1 μM or 1 mM free calcium buffers at pH 5. Beads absorbed with *CalipHluor2.0* were then imaged in DCF (G), Rhod-5F (O), and Alexa Fluor 647 (R) on a glass slide under the same exposure settings as used for measurements later. Background-subtracted G, O, and R intensities were used to calculate G/R at pH 5.1 to correct Eq. 1 and O/R_min_ and O/R_max_ to correct Eq. 3.

#### In vivo pH and calcium measurements

pH and Ca^2+^ measurements in worms were carried out as previously described for *CalipHluor_Ly_*, but with *CalipHluor2.0*. *CalipHluor2.0* is endocytosed by scavenger receptors and marks lysosomes of coelomocytes in live worms (*17*, *53*–*56*). Briefly, 500 nM *CalipHluor2.0* was microinjected into the pseudocoelom of young adult worms with the indicated genetic background. After microinjections, worms were incubated for 2 hours for maximum labeling of coelomocyte lysosomes. Worms were then anesthetized using 40 mM sodium azide in M9 solution and imaged by widefield microscopy (details in the “Image acquisition” section). The resulting images were background-subtracted before calculating the G/R and O/R ratios for each lysosome. Equation 1 was then used to calculate the pH at every lysosome. Equation 2 was then used to calculate the *K*_d_ of *CalipHluor2.0* for Ca^2+^ at every lysosome. Equation 3 was then used to calculate the O/R_min_ and O/R_max_ of *CalipHluor2.0* at every lysosome. Last, the pH-corrected free [Ca^2+^] was calculated for every lysosome using Eq. 4

$$\left[Ca^{2+}\right]=K_{d}*\left[\frac{O/R-O/R_{\text{min}}}{O/R_{\text{max}}-O/R}\right]$$ (4)

Three independent experiments, each with >5 worms, were made for pH and [Ca^2+^] values for each genetic condition. Lysosomes with O/R values below O/R_min_ were designated as having a [Ca^2+^] less than 0.1 μM. Lysosomes with O/R values above O/Rmax were designated as having a [Ca^2+^] above 1 mM. To estimate a maximum [Ca^2+^] for the exchanger-dead mutant worms, lysosomes with <0.1 μM [Ca^2+^] were counted as having 0.1uM [Ca^2+^].

#### pH and −log[Ca^2+^] maps

Images were acquired in three channels (DCF, Rhod-5F, and Alexa Fluor 647) by widefield microscopy to quantify pH and [Ca^2+^] at the single-lysosome resolution as described above. All image calculations below were done using ImageJ modules. To show representative pH and [Ca^2+^] maps, images were background-subtracted and smoothed. The Alexa Fluor 647 image was then duplicated and thresholded to create a binary mask. Background-subtracted DCF, Rhod-5F, and Alexa Fluor 647 images were then multiplied with the binary mask to get processed images. The processed DCF and Rhod-5F images were divided by the processed Alexa Fluor 647 image to get a pseudocolor G/R and O/R image, respectively. The G/R image was then plugged into Eq. 1 to get a pseudocolored pH map. The pseudocolored pH map was then used to get a pseudocolored *K*_d_ map using Eq. 2 and a pseudocolored O/R_min_ map using Eq. 3. The O/R image, *K*_d_ map, and O/R_min_ map were then used to get a [Ca^2+^] map using Eq. 4. Last, to compare maps on an appropriate calibration scale, the −log[Ca^2+^] map was calculated.

### Lysosomal pH and Ca^2+^ steady-state imaging in cells

Lysosomal pH and Ca^2+^ measurements in live mammalian cells were made using similar methods as in live worms, but with *CalipHluor^mLy^*, which contains pH sensor Oregon Green 488 (OG488) instead of DCF. Hence, the preparation and calibration of *CalipHluor^mLy^* were done using the sequences in table S2 and the equations above, but with the appropriate calibration curve for OG488, as previously described (*17*).

To make measurements in HeLa cells, the appropriate chase time was first determined by analyzing colocalization with endosomal markers. For colocalization with late endosomes, HeLa cells were transfected with Rab7-EGFP. Two days later, cells were treated with 500 nM Alexa Fluor 647-labeled double-stranded DNA (dsDNA) in Hanks’ balanced salt solution (HBSS) for 15 min before washing the dsDNA off with PBS and chasing in DMEM for the indicated amount of time (3 or 5 hours). For colocalization with lysosomes, HeLa cells were treated with FITC-dextran (5 mg/ml) in DMEM for 3 hours. Cells were then washed three times with PBS and incubated overnight in DMEM. Cells were then treated with 500 nM Alexa Fluor 647-labeled dsDNA in HBSS for 15 min before washing the dsDNA off with PBS and chasing in DMEM for the indicated amount of time (3 or 5 hours). For both colocalization experiments, cells were imaged on a confocal microscope (see the “Image acquisition” section) in the relevant channels before determining colocalization using PCC.

pH and Ca^2+^ measurements were then made by incubating HeLa cells in 500 nM *CalipHluor^mLy^* in HBSS for 15 min, washing three times with PBS, and then incubating cells in HBSS for 5 hours. Cells were then imaged on a widefield microscope (see the “Image acquisition” section) in the relevant channels. The resulting images were background-subtracted before calculating the G/R and O/R ratios for each lysosome. The above equations were used to calculate the pH and Ca^2+^ concentration for individual lysosomes. Three independent experiments, each with >50 lysosomes, were made for pH and [Ca^2+^] values for each genetic condition. Lysosomes with O/R values below O/R_min_ were designated as having a [Ca^2+^] less than 0.1 μM. Lysosomes with O/R values above O/Rmax were designated as having a [Ca^2+^] above 1 mM. Lysosomes with pH < 4 were not included in Ca^2+^ measurements. pH and −log[Ca^2+^] maps were made as described above.

### Bioinformatics

### Topology modeling

The secondary structure of human LCI was determined using JPred. Two-dimensional topology schemes of human LCI and Vcx1 were then prepared using TOPO2 software (outside residues, membrane residues, signature regions, and special residues off). TM segments were arranged to display the internal homology of human LCI and Vcx1 (tables S3 and S4), with putative Ca^2+^-binding domains forming the pore in the center. Residues are colored to show the homology of three critical regions between human LCI and Vcx1: Ca^2+^-binding region #1, yellow, 36.36% identity, 54.54% similarity; Ca^2+^-binding region #2, purple, 22.22% identity, 88.88% similarity; acidic helix, green, 31.8% identity, 50.0% similarity. Percentage identity and similarities were calculated using LALIGN (BLOSUM50 matrix; -12 gap open; -2 gap extend; 10.0E() threshold). Light blue indicates the EXGDK/R motifs in human LCI and the GNXXE motifs in Vcx1, which are involved in Ca^2+^ binding. Red indicates the lysosome-targeting YNRL motif in human LCI and the vacuole-targeting YNRV motif in Vcx.1.

#### Sequence alignments

Alignments of *VCX*, *TMEM165*, *GDT1*, and *Y54F10AL.1* sequences were performed using Clustal Omega (output format: ClustalW with character counts; mBed-like clustering; 0 combined iterations; guide tree iterations off; HMM iterations off). Select regions were highlighted based on the homology identified above and outlined in the respective colors.

### Homology modeling

Homology-based modeling of human LCI was performed using MODELLER using vcx1 as a unifying template; information about other templates is provided in table S5. The script was obtained from the MODELLER online manual provided by A. Sali using the automodel class. The resulting model was uploaded into PyMol and colored according to the previous designations.

### Cytosolic Ca^2+^ dynamics

To evaluate cytosolic Ca^2+^ dynamics, we used the ratiometric Ca^2+^ dye Fura Red according to the manufacturer’s protocols. First, HeLa (WT or *TMEM165* KO) or COS-7 cells were mock-transfected or transfected with the indicated mutant of human LCI-EGFP. Two days later, cells were pulsed with 10 μM Fura Red in HBSS for 15 min, washed three times with PBS, and then chased for 30 min in HBSS. Where indicated, 500 nM bafilomycin A was included in the chase step and every buffer afterward. Before imaging in Tyrode’s solution (134 mM NaCl, 2.68 mM KCl, 1.8 mM CaCl_2_, 1.05 mM MgCl_2_, 0.417 mM NaH_2_PO_4_, 11.9 mM NaHCO_3_, and 5.56 mM d-glucose), cells were washed three times again with PBS. The imaging protocol was set up to Fura Red images every 5 s. After eight image acquisitions, the solution was replaced with Tyrode’s solution supplemented with 100 μM ATP. Images were taken for 5 min. Fura Red images were then background-subtracted by the intensity of a region of interest (ROI) outside the cell. The 440/647 image was then duplicated and thresholded to create a binary mask. Both images were multiplied by the mask to create processed 440/647 and 488/647 images. The processed 440/647 image was then divided by the processed 488/647 image to get a 440/488 (excitation ratio) pseudocolored map. For representative images, the pseudocolored maps at indicated time points were then smoothed. The 440/488 ratio was then plotted as a function of time, normalized to 1 at *t* = 0 s.

The effectiveness of bafilomycin A at basifying lysosomal pH was evaluated with FITC-dextran. Briefly, COS-7 cells were pulsed with FITC-dextran (5 mg/ml) for 3 hours, washed three times with PBS, and incubated for 16 hours in DMEM. Cells were treated with 500 nM bafilomycin A or DMSO control (untreated, UT) for 30 min in HBSS before imaging by widefield microscopy. Cells were imaged in the 488/514 and 440/514 channels described in the “Image acquisition” section. Images were background-subtracted using the intensity of an ROI outside of the cells. The 488/514 image was thresholded to create a binary mask. Both the 488/514 and 440/514 images were multiplied by the mask to generate processed images. The processed 488/514 image was divided by the processed 440/514 image to get a 488/440 (excitation ratio) pseudocolored map and smoothed to give the representative images. The 488/440 ratio was determined for individual endosomes and plotted for each treatment condition.

### Lysosomal pH and Ca^2+^ population dynamics

To evaluate lysosomal pH population dynamics, we used the ratiometric lysosomal pH indicator FITC-dextran according to the manufacturer’s protocols. HeLa cells were pulsed with FITC-dextran (5 mg/ml) for 3 hours in DMEM before washing three times with PBS and incubating for 16 hours in DMEM. Before imaging in Tyrode’s solution, cells were washed three times again with PBS. The imaging protocol was set up to take FITC-dextran images every 5 s. After eight image acquisitions, the solution was replaced with Tyrode’s solution supplemented with 100 μM ATP. Images were taken for 5 min. FITC-dextran images were then background-subtracted by the intensity of an ROI outside the cell. The 440/514 image was then duplicated and thresholded to create a binary mask. Both images were multiplied by the mask to create processed 440/514 and 488/514 images. The processed 488/514 image was then divided by the processed 440/514 image to get a 488/440 (excitation ratio) pseudocolored map. For representative images, the pseudocolored maps at indicated time points were then smoothed. The 488/440 ratio was then plotted as a function of time, normalized to 1 at *t* = 0 s.

### Lysosomal pH and Ca^2+^ 2-IM

To evaluate the effect of human LCI on single-lysosome pH and Ca^2+^ dynamics, we used *CalipHluor^mLy^* to generate 2-IM maps before and after a cytosolic Ca^2+^ spike. To label lysosomes, WT or *TMEM165* KO HeLa cells were pulsed with 500 nM *CalipHluor^mLy^* in HBSS for 15 min, washed three times with PBS, and incubated cells in HBSS for 5 hours. Cells were then imaged on a widefield microscope (see the “Image acquisition” section) in the relevant channels before incubating cells in HBSS with 100 μM ATP. Two minutes later, cells were imaged again in the same channels. The resulting pre-ATP and post-ATP images were background-subtracted before calculating the G/R and O/R ratios for each lysosome. The above equations were used to calculate the pH and Ca^2+^ concentration for individual lysosomes before and after ATP addition. Lysosomes with O/R values above O/Rmax were designated as having a [Ca^2+^] above 1 mM. Lysosomes with pH < 4 were labeled as such, and not included in Ca^2+^ measurements. pH and −log[Ca^2+^] maps were made as described above. A lysosomal pH increase or decrease was defined as an increase or decrease, respectively, of 0.1 pH units or more. A lysosomal Ca^2+^ increase or decrease was defined as a fold change of more than 1.5 or less than 0.67, respectively.

### Whole-cell electrophysiology

Whole-cell recordings were performed with an Axopatch 200A amplifier (Molecular Devices) and digitized using a NI-6251 DAQ (National Instruments). The amplifier and digitizer were controlled using the WinWCP software (Strathclyde Electrophysiology Software). All data were sampled at 10 kHz and later filtered at 1 kHz using a four-pole low-pass Bessel filter. The borosilicate glass capillaries (Sutter) with dimensions of 1.5 mm by 0.86 mm [outer diameter/inner diameter (OD/ID)] were pulled using a Sutter P-97 micropipette puller and polished using Coater/polisher Microforge (ALA Scientific Instruments Inc.) to achieve a resistance of 2 to 4 megohms. Patch pipettes were then positioned using an MP325 motorized manipulator (Sutter). The buffers used for the bath and pipette solutions were designed to reduce background currents from ions other than calcium. The extracellular/bath solution contained the following (in millimolars): 140 NMDG, 10 Hepes, 1 MgCl_2_, 5 EGTA, 5 d-glucose, and variable Ca(OH)_2_ to get 30 nM, 300 nM, or 100 μM free [Ca^2+^], and was set to either pH 5.5, 6.5, or 7.5 with MSA. The pipette solution contained the following (in millimolars): 140 NMDG, 10 Hepes, 1 MgCl_2_, 5 EGTA, and variable Ca(OH_2_) to get either 30 nM, 100 nM, 1 μM, or 300 nM free [Ca^2+^], and was set to pH 5.5 or 7.5 with MSA. Total calcium at each pH was calculated to maintain the indicated amount of free calcium using https://somapp.ucdmc.ucdavis.edu/pharmacology/bers/maxchelator/CaMgATPEGTA-TS.htm for each experiment. Where indicated, internal Ca(OH)_2_ was replaced with Ba(OH)_2_ or MgCl_2_ to get the equivalent concentration of divalent cation. Mock-transfected or human LCI-EGFP–transfected (WT or E248A) HeLa cells were washed in PBS before incubating in the indicated extracellular solution for whole-cell clamping. Once the whole-cell configuration was established, membrane capacitance and series resistance were compensated according to established protocols (*57*). A ramp protocol (−100 to +100 mV, 1 s, holding at 0 mV) was used to record the currents. All experiments were performed at RT (22° to 23°C) and analyzed with WinWCP and OriginPro 2022b (OriginLab). As indicated in fig. S16B, positive voltage is taken as cytosol-positive, and positive current is taken as positive current moving outward from the cytosol. The current density was calculated by normalizing the current with whole-cell capacitance, which was determined immediately after establishing the whole-cell patch to estimate cell surface area. The average traces shown were smoothed using seven-point adjacent averaging. Where indicated, the current density at +100 or −100 mV was plotted for each condition. Theoretical reversal potentials were calculated as previously described (*58*).

### Lysosome electrophysiology

Borosilicate glass capillaries (Sutter) with dimensions of 1.5 mm by 0.86 mm (OD/ID) were pulled using the following program: heat, 520; pull, 0; vel, 20; time, 200; loops, 4. Pipettes were fire-polished using an MF200 microforge (World Precision Instruments) and used for voltage clamping. For fig. S18 (D and E), the pipette and bath solutions mimicked the ionic composition of the lysosome (pH 4.5) or Golgi (pH 6.2) and cytoplasm, respectively (see fig. S18C). The cytosolic/bath solution contained the following (in millimolars): 20 KCl, 120 K-gluconate, 2 MgCl_2_, 2.5 CaCl_2_, 0.2 EGTA, 10 Hepes, and 2 Na_2_ATP (pH 7.25). The pipette/lumenal solution contained the following (in millimolars): 145 NaCl, 20 d-glucose, 5 Na_3_Cit, 10 EGTA, 2 CaCl_2_, 1 MgCl_2_, 10 TEA, and 3 KCl (pH 4.5 or 6.2). Mock-transfected or human LCI-EGFP–transfected COS-7 cells were treated with 5 μM vacuolin-1 to swell lysosomes to 1 to 3 μm (*59*). Cells were washed three times with PBS and incubated in the indicated bath solution, cells ruptured, and enlarged lysosomes pushed out. After gigaohm seal formation, the break-in was performed by a zap protocol (5 V: 0.5 to 5 s) until the appearance of capacitance transients. Voltage ramping and calculation of current were performed as described above. Positive membrane potential is taken as cytosol-positive, and positive current is taken as positive current moving outward from the cytosol. Representative traces were smoothed by 24-point adjacent averaging and current at +100 mV was plotted for each condition.

### Image acquisition

Widefield microscopy was carried out on an IX83 inverted microscope (Olympus Corporation of the Americas, Center Valley, PA, USA) using a 60×, 1.4 numerical aperture (NA), differential interference contrast oil immersion objective (PLAPON) and Evolve Delta 512 EMCCD camera (Photometrics, USA), and controlled using MetaMorph Premier Ver 7.8.12.0 (Molecular Devices, LLC, USA). For *CalipHluor2.0* imaging in worms, images were acquired with an exposure of 150 ms and an EM gain of 150 for DCF, an exposure of 150 ms and an EM gain of 150 for Rhod-5F, and an exposure of 50 ms and an EM gain of 50 for Alexa Fluor 647. DCF channel images were obtained using a 480/20 band-pass excitation filter, a 520/40 band-pass emission filter, and an 89016 dichroic; Rhod-5F channel images were obtained using a 545/25 band-pass excitation filter, a 595/50 band-pass emission filter, and an 89016 dichroic; and Alexa Fluor 647 channel images were obtained using a 640/30 band-pass excitation filter, a 705/72 band-pass emission filter, and an 89016 dichroic. For *CalipHluor*^*mLy*^ imaging in cells, images were acquired with an exposure of 200 ms and an EM gain of 200 for OG488, an exposure of 200 ms and an EM gain of 200 for Rhod-5F, and an exposure of 200 ms and an EM gain of 200 for Alexa Fluor 647. OG488 channel images were obtained using a 480/20 band-pass excitation filter, a 520/40 band-pass emission filter, and an 89016 dichroic; Rhod-5F channel images were obtained using a 545/25 band-pass excitation filter, a 595/50 band-pass emission filter, and an 89016 dichroic; and Alexa Fluor 647 channel images were obtained using a 640/30 band-pass excitation filter, a 705/72 band-pass emission filter, and an 89016 dichroic. For cytosolic Ca^2+^ recording, Fura Red was recorded by excitation at 440 or 488 nm and emission at 647 nm. The 440/647 images were acquired using a 430/24 band-pass excitation filter, a 705/72 band-pass emission filter, and an 89016 dichroic with an exposure of 150 ms and an EM gain of 150. The 488/647 images were acquired using a 480/20 band-pass excitation filter, a 705/72 band-pass emission filter, and an 89016 dichroic with an exposure of 200 ms and an EM gain of 200. For lysosomal pH recording in cells, FITC-dextran was recorded by excitation at 440 or 488 nm and emission at 514 nm. The 440/514 images were acquired using a 430/24 band-pass excitation filter, a 520/40 band-pass emission filter, and an 89007 dichroic with an exposure of 200 ms and an EM gain of 200. The 488/514 images were acquired using a 480/20 band-pass excitation filter, a 520/40 band-pass emission filter, and an 89016 dichroic with an exposure of 200 ms and an EM gain of 200. EGFP images were acquired using a 480/20 band-pass excitation filter, a 520/40 band-pass emission filter, and an 89016 dichroic. DsRed images were acquired using a 545/25 band-pass excitation filter, a 595/50 band-pass emission filter, and an 89016 dichroic. FM4-64 images were acquired using a 545/25 band-pass excitation filter, a 632/60 band-pass emission filter, and an 89016 dichroic.

Confocal images were captured with a Leica TCS SP5 II STED laser confocal microscope (Leica Microsystems, Buffalo Grove, IL, USA) equipped with a 63×, 1.4 NA, oil immersion objective. GFP and Alexa Fluor 488 were excited using an argon laser with a wavelength of 488 nm; Alexa Fluor 647 was excited using a He-Ne laser with a wavelength of 633 nm; and TMR, DsRed, and Alexa Fluor 555 were excited using a DPSS laser at 561 nm. All emissions were filtered using an acousto-optical beam splitter with settings suitable for each fluorophore and recorded using the hybrid detector.

### Image analysis

Images were analyzed using Fiji (NIH, USA). For lysosomal pH and Ca^2+^ measurements with *CalipHluor 2.0*, coelomocytes containing single lysosomes in each Alexa Fluor 647 (R) image were manually selected and coordinates were saved in the ROI plugin. The most focused plane was manually selected in the Alexa Fluor 647 channel and used for all other channels. After background subtraction, the mean intensity for each endosome (G, O, and R) was measured and exported to OriginPro (OriginLab, USA). Ratios of G to R (G/R) and O to R (O/R) intensities were then obtained. Representative images are shown as pseudocolored maps, where G, O, and R images were modified by thresholding in ImageJ. Lysosomal Ca^2+^ maps with *CalipHluor^mLy^*, lysosomal pH maps with FITC-dextran, and cytosolic Ca^2+^ maps with Fura Red were similarly made.

### Statistics

For statistical analysis between two samples, a two-sample two-tailed test assuming unequal variance was conducted. For the comparison of multiple samples, a one-way analysis of variance (ANOVA) with a post hoc Tukey test was conducted. All statistical analysis was performed in OriginLab.

## Supplementary Materials

### This PDF file includes:

Supplementary Text S1 to S4 Figs. S1 to S18 Tables S1 to S6 References
