# Supplementary material for: A mechanism of lysosomal calcium entry
Source: Sci Adv. 2024 Feb 14;10(7):eadk2317. doi: 10.1126/sciadv.adk2317 (PMC10866540; doi:10.1126/sciadv.adk2317)
Supplement: Supplementary file 1 — Supplementary Text S1 to S4 Figs. S1 to S18 Tables S1 to S6 References [file sciadv.adk2317_sm.pdf]

Science Advances
AAAS

Supplementary Materials for

A mechanism of lysosomal calcium entry

Matthew Zajac *et al.*

Corresponding author: Anand Saminathan, anand.saminathan@childrens.harvard.edu;
Yamuna Krishnan, yamuna@uchicago.edu

Sci. Adv. **10**, eadk2317 (2024)
DOI: 10.1126/sciadv.adk2317

The PDF file includes:

Supplementary Text S1 to S4
Figs. S1 to S18
Tables S1 to S6
References

Supplementary Text

1. Localization of WT LCI

Our data show that WT human LCI is largely localized to the Golgi in HeLa cells, with minimal colocalization with TMR-dextran. However, previous experiments have found human LCI in lysosome fractions (*24*). In addition, we have detected overexpressed and endogenous human LCI on membranes of lysosomes (**Fig. S4, S18A,B).** Even a small fraction of human LCI present on the lysosomes would be highly active, given the higher pH gradient there than across the Golgi membrane. Thus, the low lysosome localization does not preclude human LCI from having a physiologically relevant role on the lysosome membrane.

2. Lysosomal calcium measurements

The O/R ratio of ~50% of lysosomes of *lci-1*$^{+/-}$ worms was below the O/R_{min} of *CalipHluor2.0*, indicating a Ca^{2+} concentration <100 nM that is not quantifiable by our probe (**Fig. S6D**). Conversely, the O/R ratio of ~50% of lysosomes of *lci-1*$^{+/-}$ worms expressing WT human LCI was above the O/R_{max} of *CalipHluor2.0,* indicating a Ca^{2+} concentration >1 mM that is not quantifiable by our probe (**Fig. S6D**). Thus, our reported effect of human LCI on lysosomal Ca^{2+} levels in worms is actually an underestimation.

Similarly, the O/R ratio of over 60% of lysosomes of TMEM165 KO HeLa cells was below the O/R_{min} of *CalipHluor*^{mLy} (**Fig. S13C)**. Only about 30% of lysosomes of WT HeLa cells had an O/R below the O/R_{min} (**Fig. S13C**). Thus, the reported effect of human LCI on lysosomal Ca^{2+} levels in cells is also an underestimation.

The lysosomal Ca^{2+} measurements of worms expressing mutants of human LCI are complicated by the heterozygous knockout background. Specifically, we see a surprisingly high level of lysosomal Ca^{2+} in *lci-1*$^{+/-}$ worms expressing the G304R, E108A, and E248A mutants of

human LCI. Yet, in all other assays, these mutants impair lysosomal Ca^{2+} import. Thus, it is likely that human LCI acts as a dimer, and that the remaining copy of endogenous worm *lci-1* can dimerize with the mutant human LCI, and form a partially functional transporter. Given the smaller size of human LCI compared to other Ca^{2+} transporters and exchangers, we hypothesize that it acts as a dimer.

3. Homologous regions of human LCI

The regions of human LCI that show homology to vcx1 offer clues to how human LCI may transport Ca^{2+} with high capacity, dependent on the lysosomal pH gradient. In vcx1, the proton motive force across the vacuole drives a conformational change where active site glutamate residues face the cytosol and maintain a negative charge (*29*). Under conditions of high cytosolic Ca^{2+}, as seen in our experiments in **Fig. 3C** in yeast and **Fig. 4 and S12A** in mammalian cells, Ca^{2+} ions are coordinated by the cytosolic acidic helix to bring them near the active site. Coordination by the active site displaces water molecules to move helix M2b (designated in yellow in **Fig. 3A,B**) towards the active site. This movement closes the cytosolic vestibule lined by M7b (designed in purple in **Fig. 3A,B**) and opens a vacuolar cleft, such that the acidic pH of the vacuole lowers the Ca^{2+} affinity of active site glutamate residues and leads to release of Ca^{2+}. This cyclical pumping occurs because of flexible helices around the active site and more rigid piston-like helices further away from the pore. Given that human LCI possesses two Ca^{2+}-binding sequences near regions homologous to the flexible internal helices of vcx1 and an acidic cytosolic helix in proximity, it follows that human LCI functions similarly in response to high cytosolic Ca^{2+}. However, the fact that human LCI is much smaller than vcx1 and most other exchangers may indicate that it functions differently, for example as a dimer or as a pH-activated transporter instead of an exchanger.

4. Yeast color change

Strains of *S. cereivisiae* that have mutations in certain steps of the adenine biosynthetic pathway (such as the ade2-1 mutation in K665 (*60*)) accumulate an adenine-intermediate-derived red pigment inside vacuoles (*61*). The intermediate phosphoribosylaminoimidazole (AIR) is transported glutathione-dependently into vacuoles where it is polymerized and modified to form the characteristic red pigment. The structure of this pigment has yet to be fully established. Importantly, development of red pigmentation in ade2 mutants requires normal vacuolar function (*62*). This concept has even been used to screen for chemicals that disrupt vacuolar function by loss of red pigmentation (*63*). Interestingly, we see that K665 colonies grown on SD-Leu plates do not exhibit red pigmentation, but that human LCI-transformed K665 colonies appear red. This red pigmentation is lost when plated on plates with high Ca^{2+}. This implies that human LCI rescues vacuolar dysfunction in K665 under normal osmotic conditions, but that high Ca^{2+} causes vacuolar dysfunction even as human LCI rescues lethality. While we cannot rule out the effect of human LCI on other aspects of the adenine-derived pigment biosynthetic pathway, its lysosomal roles in humans and nematodes established elsewhere in this manuscript support the hypothesis that it rescues vacuolar dysfunction here.

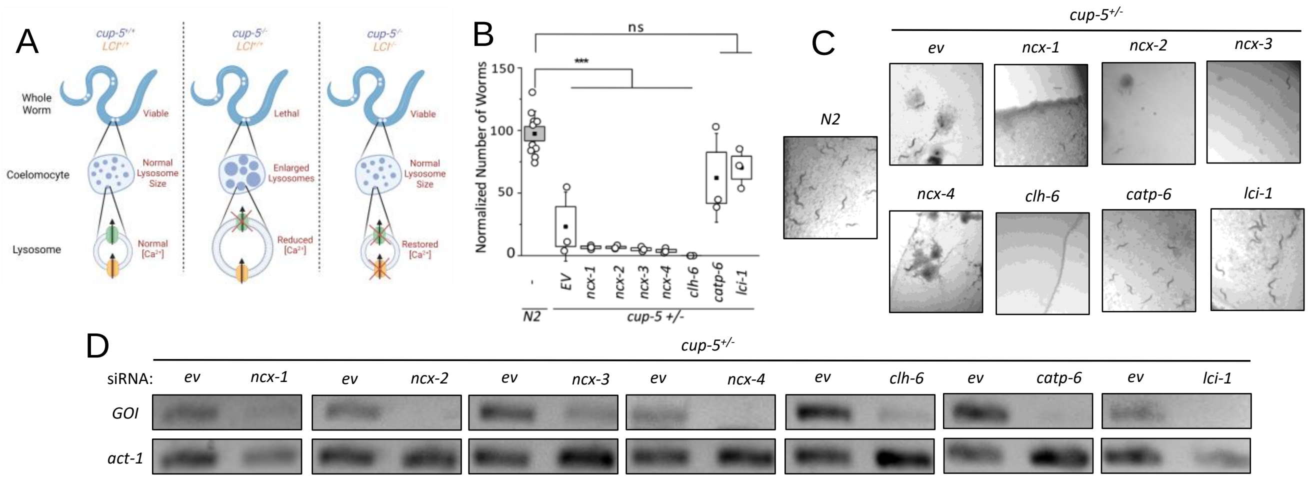

Figure S1: The activity of *lci-1* complements *cup-5*. (a) Schematic of the principle underlying screening for lysosomal calcium importers (LCIs) in cup-5$^{+/-}$ worms by survival and lysosome size. (b) Number of *cup-5*$^{+/-}$ progeny following RNAi knockdown of indicated transcripts. (c) Representative images showing the number of progeny of N2 worms or *cup-5*$^{+/-}$ worms in plates containing RNAi bacteria of empty vector (EV, control), *ncx-1*, *ncx-2*, *ncx-3*, *ncx-4*, *clh-6*, *lci-1*, or *catp-6* (positive control). (d) RT-PCR analysis of total RNA isolated from *cup-5*$^{+/-}$ worms for the indicated gene of interest (GOI) following knockdown of the indicated gene, compared to treatment with empty vector.

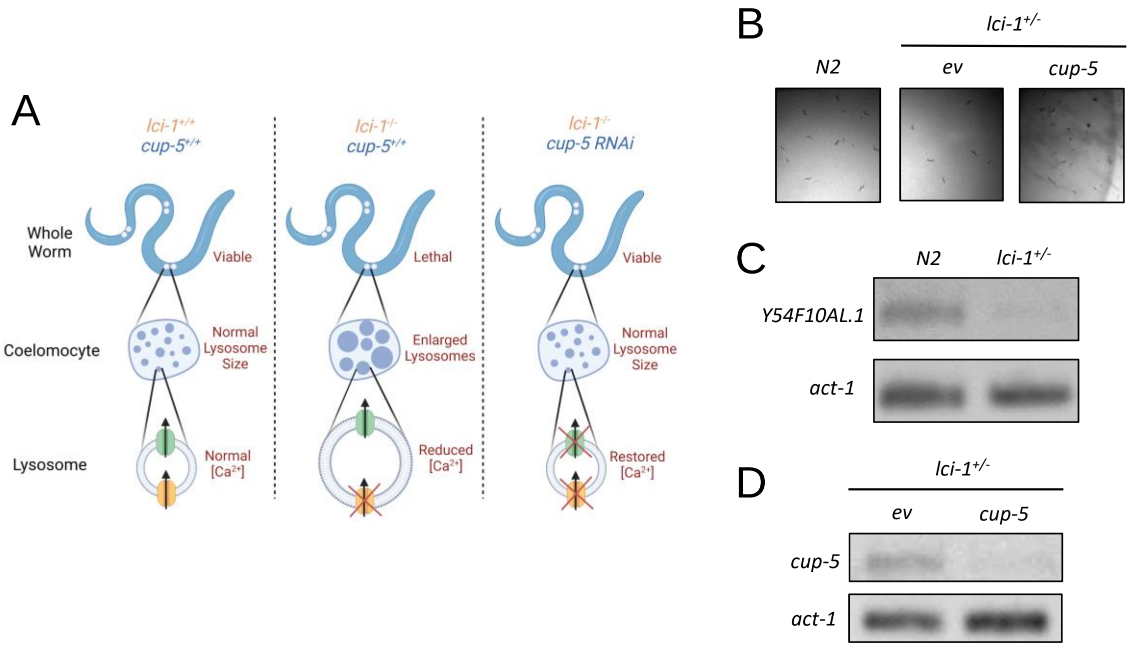

Figure S2: The activity of *cup-5* complements *lci-1*. (a) Schematic of principle behind phenotypes observed in *lci-1*$^{+/-}$ worms. (b) Representative images showing the number of progeny of N2 worms or *lci-1*$^{+/-}$ worms in plates containing RNAi bacteria of empty vector (ev, control) or *cup-5*. (c) RT-PCR analysis of total RNA isolated from N2 or *lci-1*$^{+/-}$ worms for the *lci-1* (*Y54F10AL.1)* gene, with actin used as a control. (d) RT-PCR analysis of total RNA isolated from *lci-1*$^{+/-}$ worms for the *cup-5* gene following knockdown of *cup-5*, compared to treatment with empty vector.

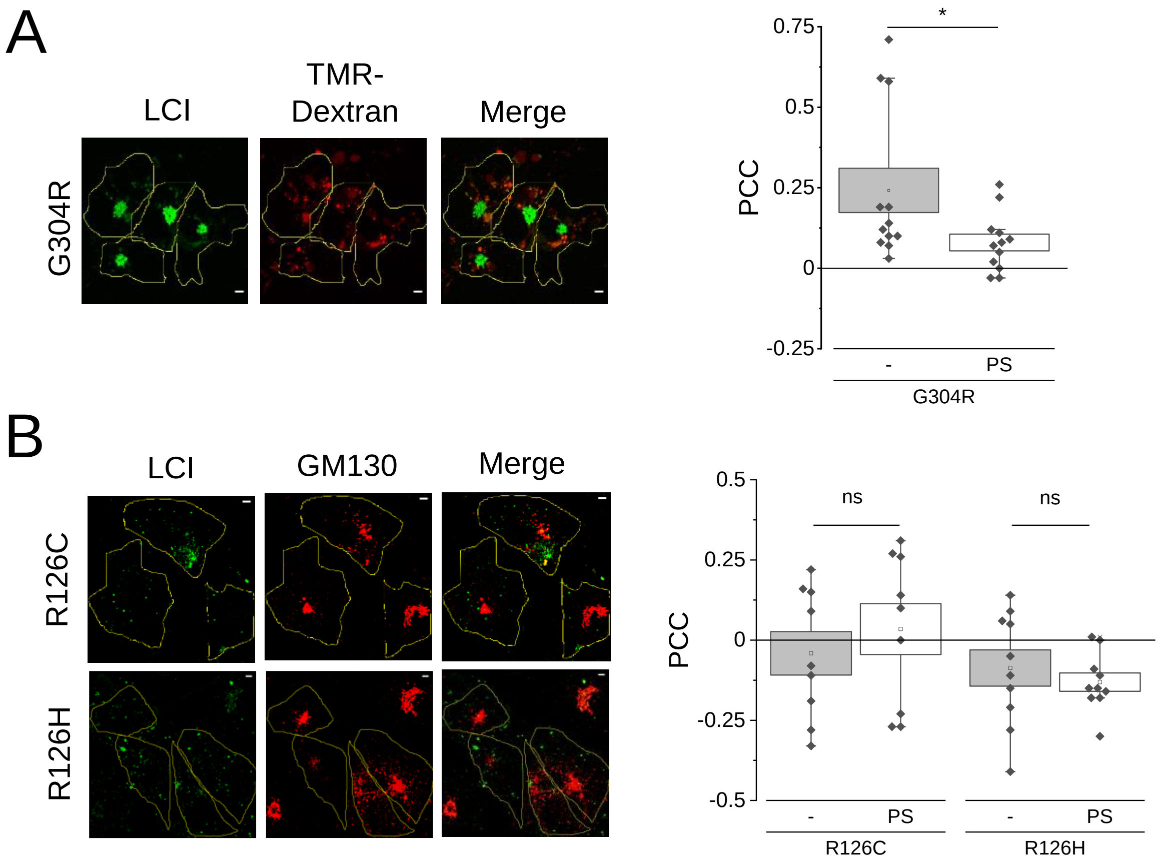

Figure S3: Localization of human LCI disease mutants. (a) Left, representative fluorescence images of COS-7 cells transiently expressing G304R human LCI-EGFP (green) and labeled with TMR-dextran (red). Right, Pearson correlation coefficient (PCC) of colocalization between G304R human LCI and TMR-dextran before and after pixel shift (PS). (b) Left, representative fluorescence images of COS-7 cells transfected with the indicated mutant of human LCI and immunostained for human LCI (green) and GM130 (red). Right, pearson correlation coefficient (PCC) of colocalization between human LCI mutants and GM130 before and after pixel shift (PS). Scale bar 5 μm. Boxes and bars represent the s.e.m. and outliers, respectively. ns, not significant ($P>0.05$); *$P<0.05$; **$P<0.01$; ***$P<0.001$.

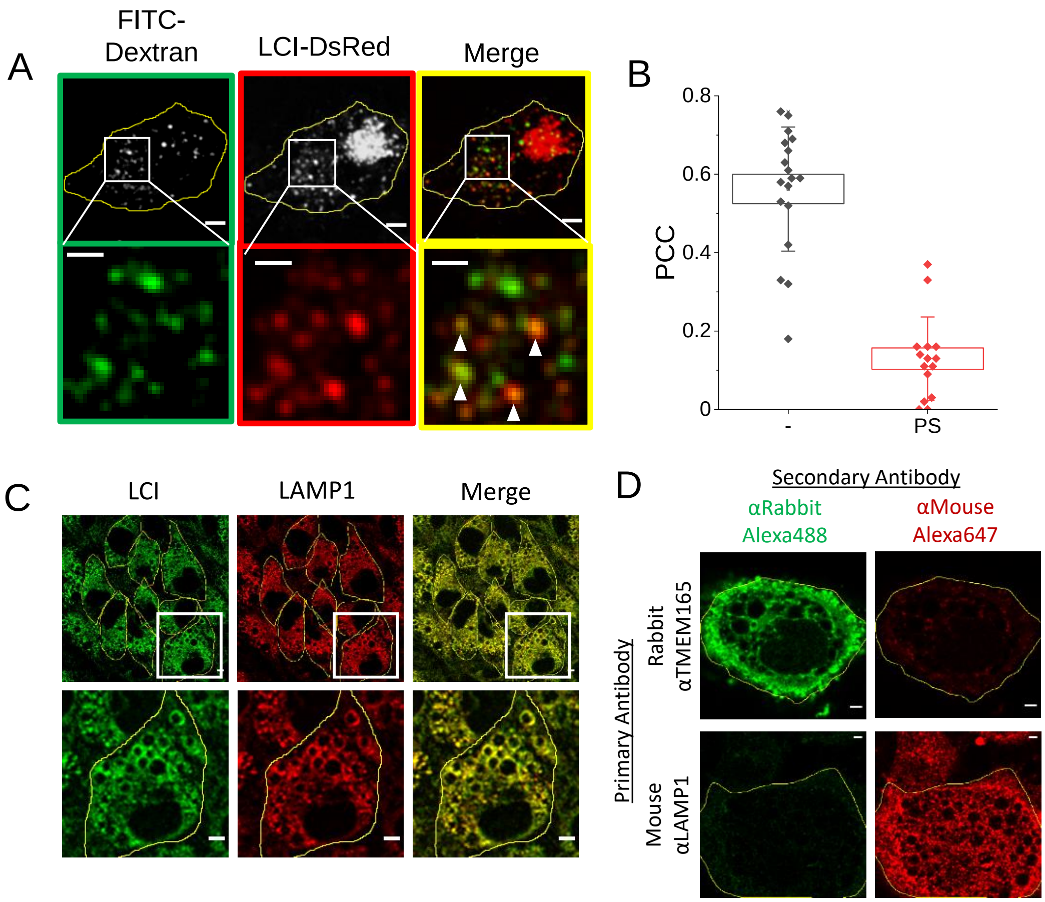

Figure S4: Lysosomal localization of human LCI. (a) Representative fluorescence images of COS-7 cells transiently expressing human LCI-DsRed (red) and labeled with FITC-dextran (green). White arrows indicate human LCI-positive lysosomes in inset merge image. (b) Pearson correlation coefficient (PCC) of colocalization between human LCI and FITC-dextran before and after pixel shift (PS). (c) Representative immunofluorescence images showing endogenous localization of human LCI in HeLa cells pre-treated with vacuolin-1 to swell lysosomes. Cells were stained with antibodies to human LCI (green) and LAMP1 (red) after glyoxal fixation to maintain lysosome structure. (d) Representative immunofluorescence images showing specificity of secondary antibodies and spectral separation of fluorophores used in (c). Scale bar 5 µm. Inset scale bar 2um. Boxes and bars represent the s.e.m. and standard deviation, respectively.

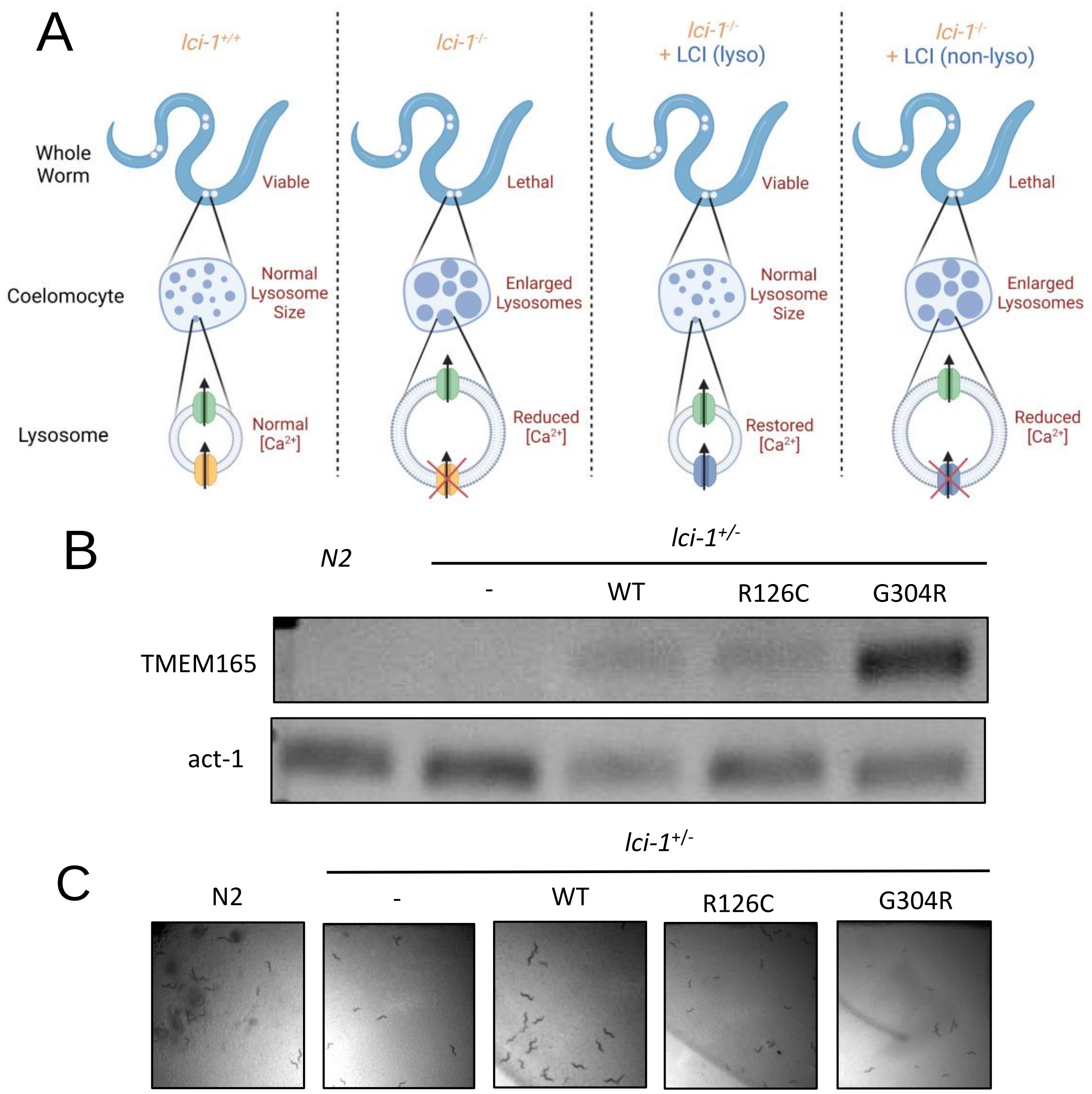

Figure S5: Human LCI rescues organism and cellular phenotypes seen in *lci-1*$^{+/-}$ worms. (a) Schematic of principle underlying rescue of *lci-1*$^{+/-}$ worm phenotypes with human LCI variants. (b) Representative images showing the number of progeny of N2 worms or *lci-1*$^{+/-}$ worms with extrachromosomal expression of the indicated mutant of human LCI. (c) RT-PCR analysis of the human LCI (TMEM165) expression in N2 worms and *lci-1*$^{+/-}$ worms with extrachromosomal expression of the indicated mutant of human LCI.

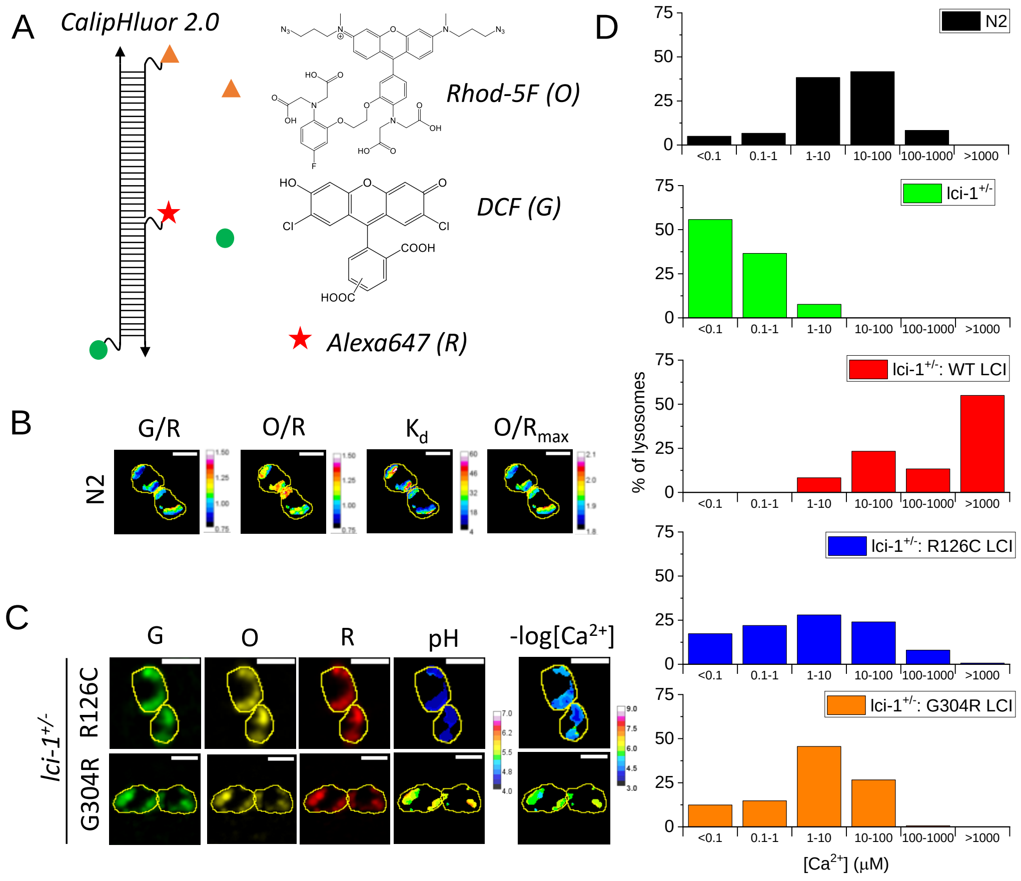

Figure S6: Human LCI rescues lysosomal Ca^{2+} dysregulation in *lci-1*$^{+/-}$ worms. (a) Schematic of *CalipHluor 2.0*, which consists of Rhod-5F (orange triangle), DCF (green circle), and Alexa647 (red star) on a DNA duplex. (b) Representative pseudocolored maps of the DCF/Alexa647 ratio (G/R) and Rhod-5F ratio (O/R) of *CalipHluor2.0* in N2 worms. These maps are used according to equations in the Methods section to generate K_d and O/R_{max} maps. (c) Representative fluorescence images and pH and -log([Ca^{2+}]) maps in *CalipHluor2.0*-labeled lysosomes in coelomocytes in *lci-1*$^{+/-}$ worms extrachromosomally expressing the indicated variant of human LCI. G, DCF; O, Rhod-5F; R, Alexa647. Scale bar 5 µm (d) Distribution of lysosomes with the indicated Ca^{2+} concentration measured using *CalipHluor2.0* in the indicated genetic backgrounds. Scale bar 5 µm.

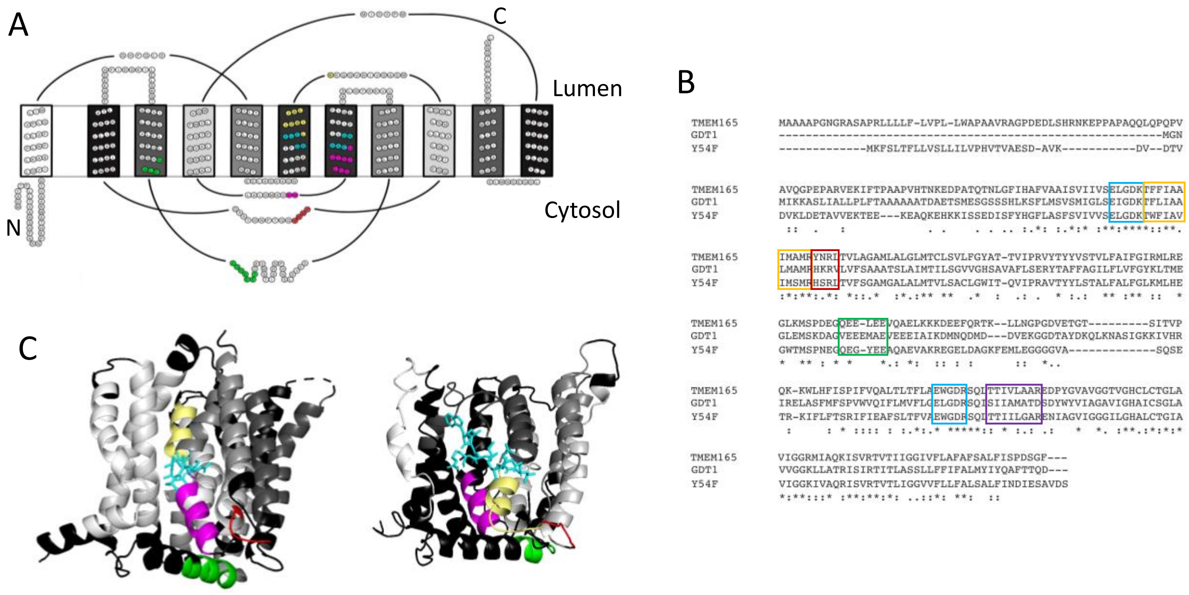

Figure S7. Conservation within the UPF0016 family and homology-based model of human LCI. (a) Topology of vcx1 created using TOPO2. Grayscale shows internal symmetry of transmembrane segments. Ca^{2+} binding regions (light blue), homologous regions adjoining the Ca^{2+} binding regions (yellow and purple), acidic helices (green), and lysosome/vacuole-targeting motifs (red) are highlighted. (b) Full sequence alignment of three proteins in the UPF0016 family: TMEM165 (*H. sapiens*), Y54F10AL.1 (*C. elegans*), and gdt1 (*S. cerevisiae*) using Clustal Omega. Asterisks indicate full conservation, colons indicate strong conservation, and periods indicate weak conservation. Calcium binding regions, homologous regions to vcx1, acidic helices, and lysosome/vacuole-targeting motifs are highlighted according to colors indicated in Figure 3. (c) Homology-based model of human LCI (right) based on vcx1 (left), with colors highlighted according to Figure 3 indications.

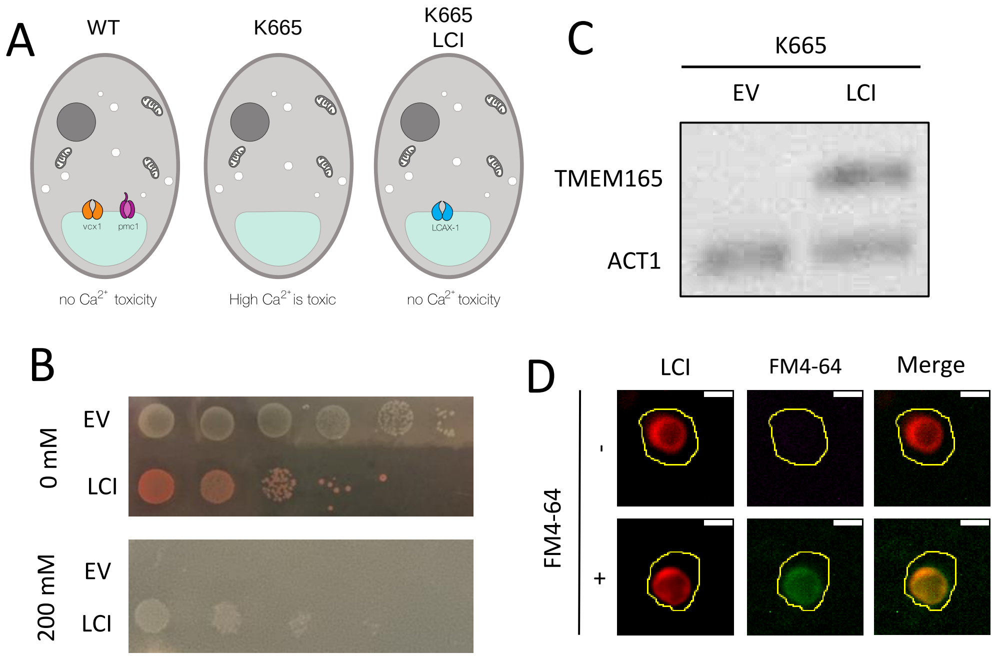

Figure S8. Human LCI rescues phenotypes of the K665 strain. (a) The rescue assay in *S. cerevisiae* strain K665, where the loss of vcx1 and pmc1 cause lethality in environmental Ca^{2+}. Expression of a vacuolar Ca^{2+} importer would rescue this lethality at high Ca^{2+} by restoring vacuolar function. (b) Color of K665 transformed to integrate an empty vector (EV) or human LCI, after 2 days at 30°C on YPD plates supplemented with the indicated concentration of $CaCl_2$. Columns indicated 10-fold dilutions from left-to-right. (c) RT-PCR of TMEM165 (*H. sapiens*) and ACT1 (*S. cerevisiae*) in the K665 strain transformed with empty vector (EV) or human LCI. (d) Representative fluorescence images of K665 transformed with human LCI-DsRed (red) and labelled with the vacuolar membrane marker FM4-64 (green), where indicated. Scale bar 2µm.

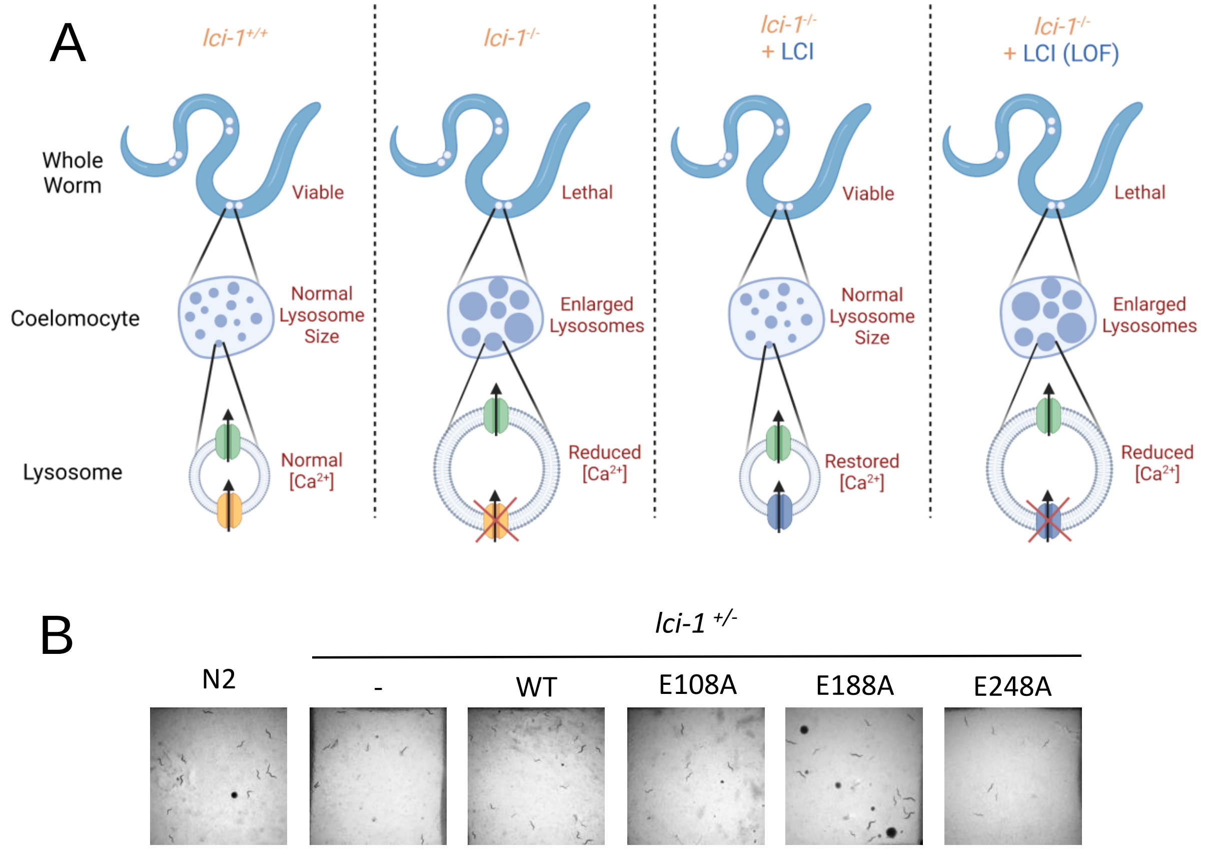

Figure S9. Functional human LCI mutants rescuing organism and cellular phenotypes seen in *lci-1*$^{+/-}$ worms (a) Schematic of principle underlying rescue of *lci-1*$^{+/-}$ worm phenotypes with human LCI loss-of-function (LOF) variants. (b) Representative images showing the number of progeny of N2 worms or *lci-1*$^{+/-}$ worms with extrachromosomal expression of the indicated mutant of human LCI.

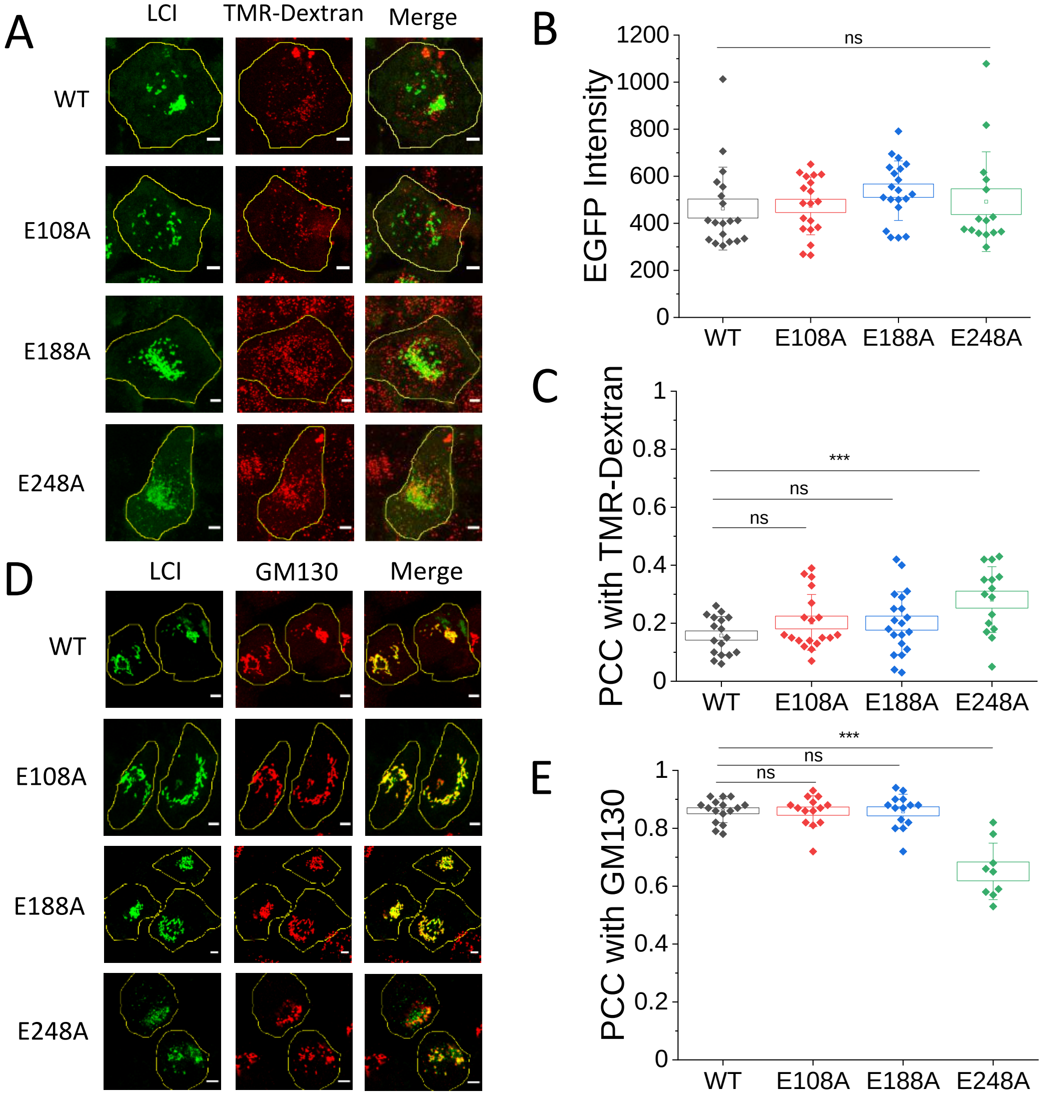

Figure S10. Expression and localization of human LCI mutants. (a) Representative fluorescence images of HeLa cells transfected with the indicated human LCI mutant (green) and labeled with 5 mg/mL TMR-dextran (red). (b) Whole-cell fluorescence intensity of human LCI-EGFP in cells transfected with the indicated mutant. (c) Pearson correlation coefficient (PCC) of human LCI-EGFP with TMR-dextran in cells transfected with the indicated mutant. (d) Representative immunofluorescence images of HeLa cells transfected with the indicated human LCI mutant (green) and stained with an antibody to GM-130 (red). (e) Pearson correlation coefficient (PCC)

of human LCI-EGFP with GM130 in cells transfected with the indicated mutant. Scale bar 5μm. Boxes and bars represent s.e.m. and standard deviation, respectively. ns, not significant (p>0.05); *p<0.05; **p<0.01; ***p<0.001 (one-way ANOVA with Tukey post hoc test).

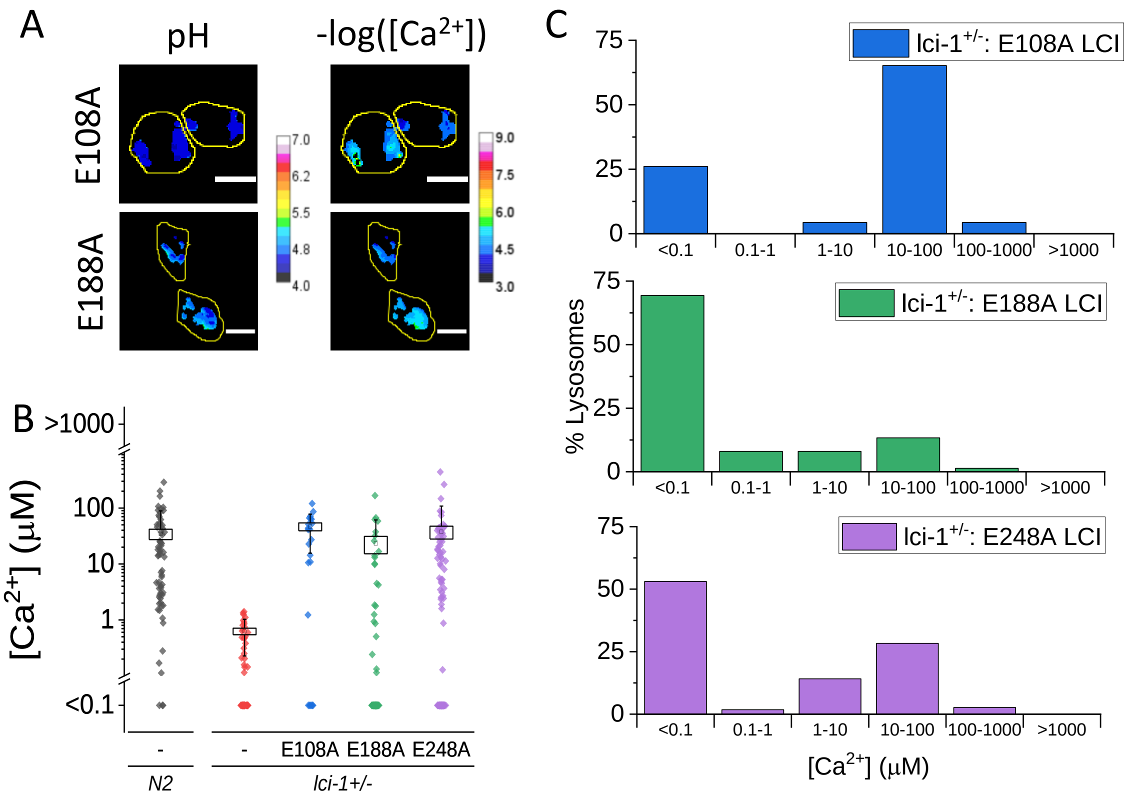

Figure S11. Lysosomal Ca^{2+} measurements in *lci-1*$^{+/-}$ worms expressing mutants of human LCI. (a) Representative fluorescence pH and -log([Ca^{2+}]) maps in *CalipHluor2.0*-labeled lysosomes in coelomocytes in *lci-1*$^{+/-}$ worms extrachromosomally expressing the indicated variant of human LCI. (b) Maximum average lysosomal Ca^{2+} concentration in the indicated genetic backgrounds. Lysosomes with <0.1µM [Ca^{2+}] are considered to have 0.1µM [Ca^{2+}]. (c) Distribution of lysosomes with the indicated Ca^{2+} concentration measured using *CalipHluor2.0* in the indicated genetic backgrounds. Scale bar 5µm. Boxes and bars represent the s.e.m. and standard deviation of lysosomes with calcium levels in the quantifiable range.

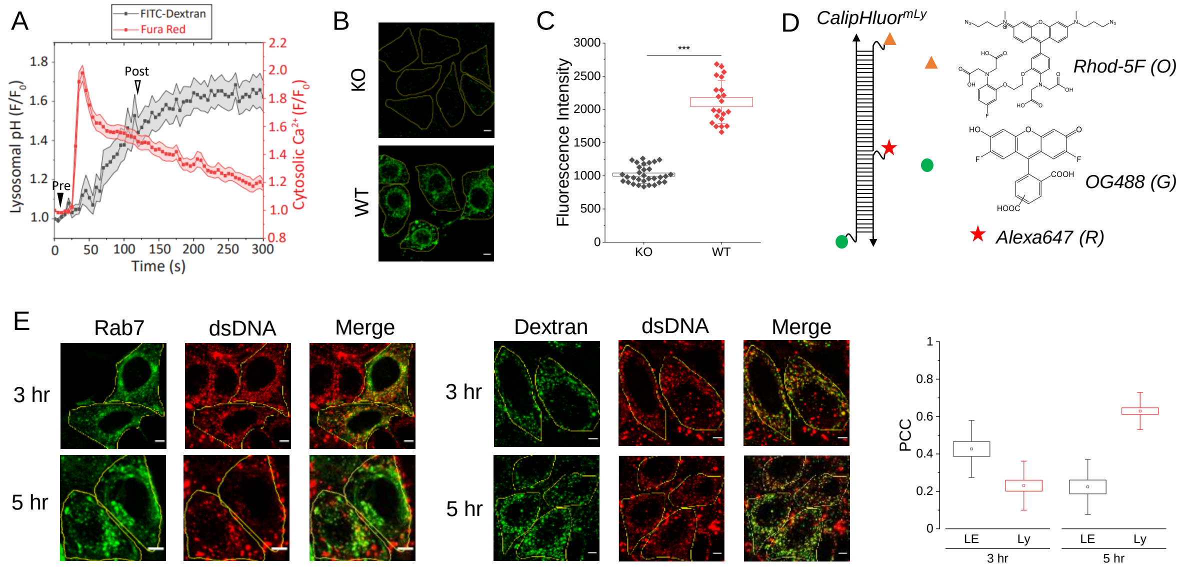

Figure S12: Justification and technology underlying single-lysosome Ca^{2+} measurements. (a) Overlay of the cytosolic Ca^{2+} recovery and lysosomal pH spike, given by the 440/488 ratio of Fura Red and the 488/440 ratio of FITC-dextran, respectively, in WT cells. Arrowheads indicate imaging time points before ATP addition (Pre, filled arrow) and after ATP addition (Post, unfilled arrow) in Figure 4. (b) Representative immunofluorescence images showing endogenous expression of human LCI in TMEM165 KO HeLa cells and WT HeLa cells. (c) Background-subtracted whole-cell fluorescence intensity of Alexa488-conjugated secondary antibody. Each data point represents the average fluorescence intensity of one cell. (d) Schematic of *CalipHluor^{mLy}*, which consists of Rhod-5F (orange triangle), Oregon Green 488 (green circle), and Alexa647 (red star) on a DNA duplex. (e) Left and middle, representative fluorescence images of HeLa cells pulsed with Alexa647-labeled dsDNA for 15 minutes and chased for the indicated amount of time. To look at late endosomal localization, cells were transfected with Rab7-GFP prior to dsDNA labeling (left). To look at lysosomal localization, cells were pulsed with FITC-dextran for 1 hour and chased overnight prior to dsDNA labeling (middle). Right,

Pearson correlation coefficient (PCC) of dsDNA with endolysosomal markers at the indicated time points. Scale bar 5 μm. ***$p<0.001$.

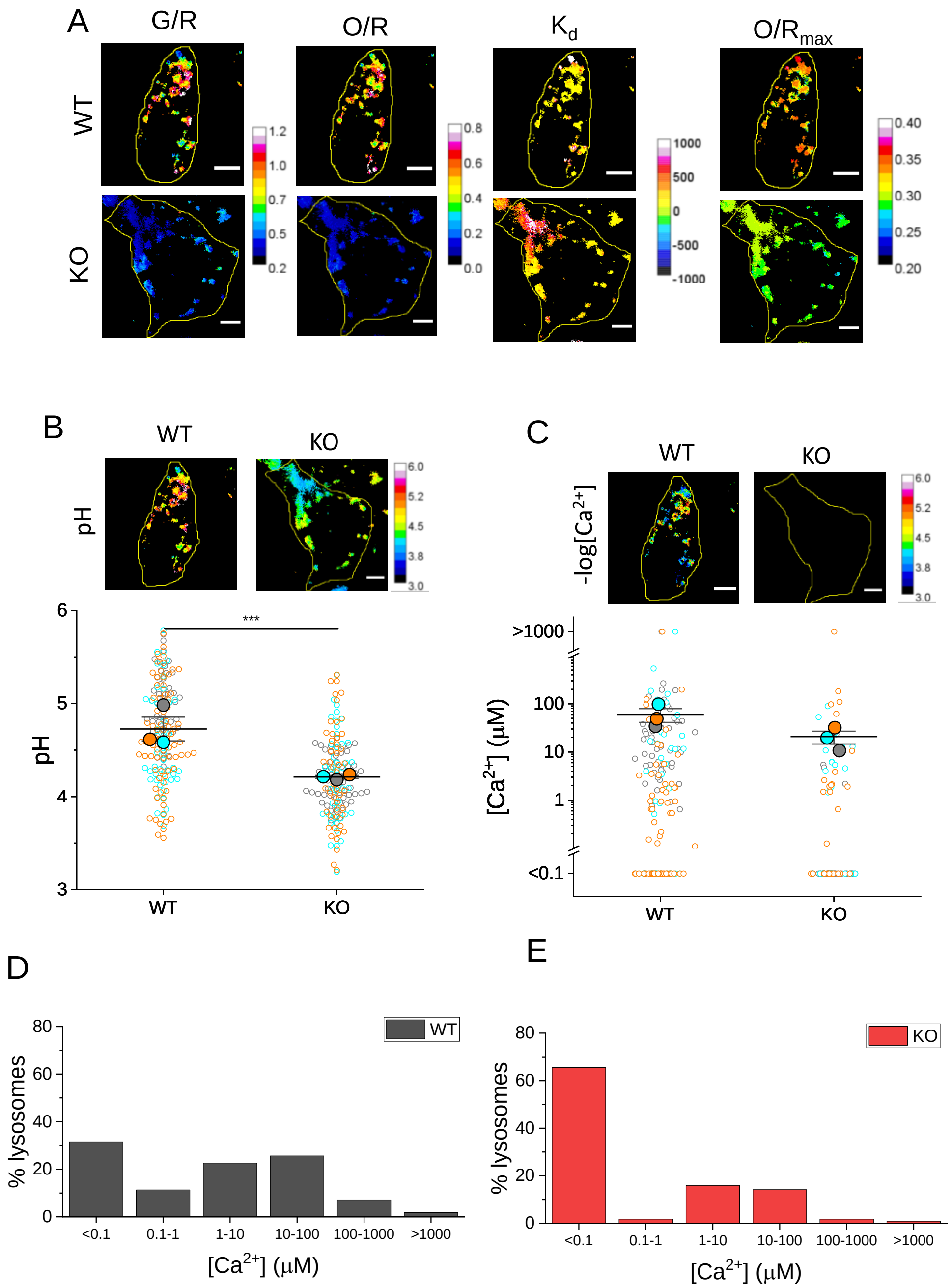

Figure S13. Reduction of lysosomal Ca^{2+} in TMEM165 KO mammalian cells. (a) Representative pseudocolored maps of the DCF/Alexa647 ratio (G/R) and Rhod-5F ratio (O/R) of *CalipHluor2.0* in WT and TMEM165 KO HeLa cells. These maps are used according to equations in the Methods section to generate K_d and O/R_{max} maps. (b) Top, representative pH maps of lysosomes from WT

and TMEM165 KO HeLa cells using *CalipHluor*^{mLy}. Bottom, pH of individual lysosomes (open circles) from three different experiments (closed circles). (c) Top, representative –log([Ca^{2+}]) maps of lysosomes from WT and TMEM165 KO HeLa cells using *CalipHluor*^{mLy}. Bottom, [Ca^{2+}] of individual lysosomes (open circles) from three different experiments (closed circles). Lysosomes below O/R_{min} or above O/R_{max} of *CalipHluor*^{mLy} are shown as $<0.1\mu m\ Ca^{2+}$ and $>1000\mu m\ Ca^{2+}$, respectively. (d) Distribution of lysosomes with the indicated Ca^{2+} concentration measured using *CalipHluor2.0* in the indicated genetic backgrounds. Scale bar 5 μm.

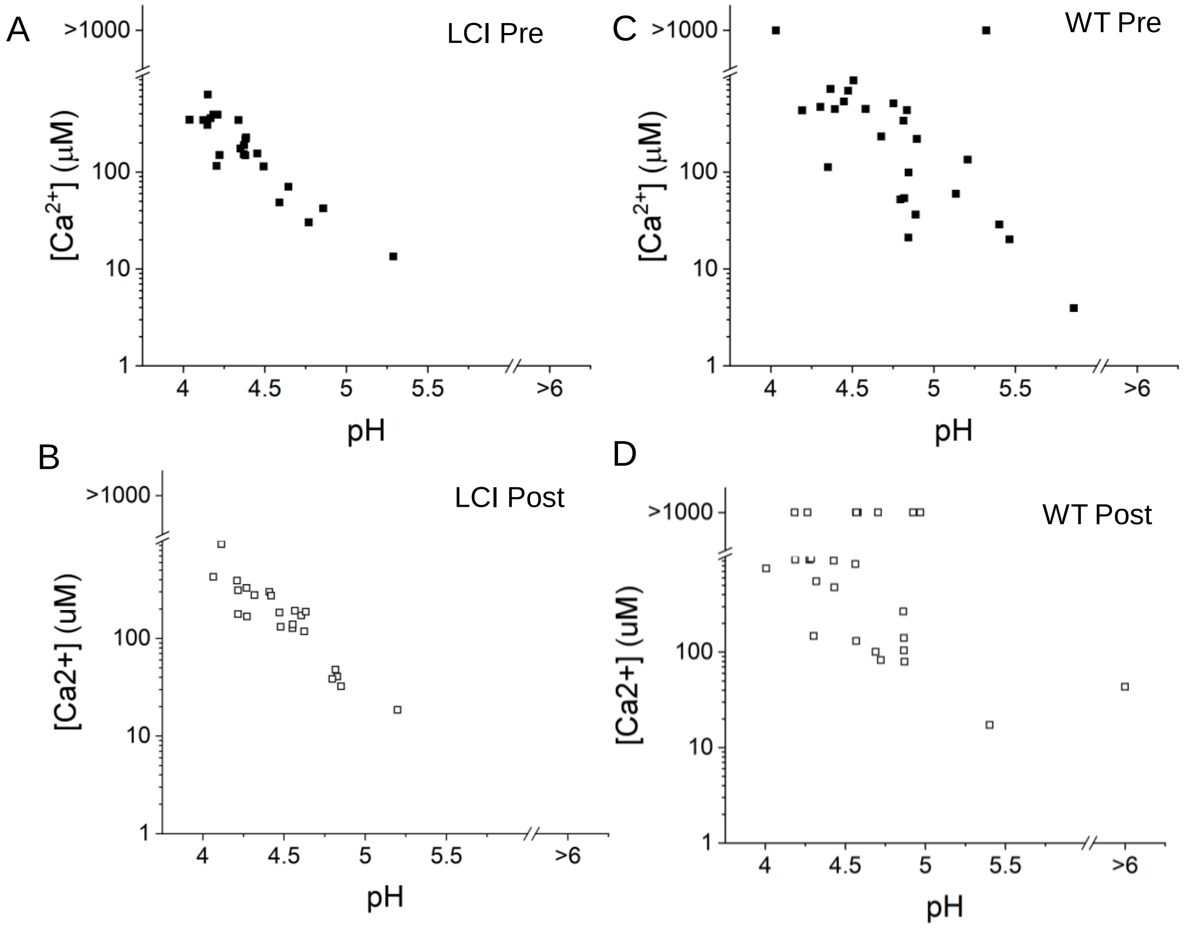

Figure S14. Individual 2-IM maps of lysosomal Ca^{2+} and pH in TMEM165 KO HeLa cells before (a) and after (b) ATP addition, and in WT HeLa cells before (c) and after (d) ATP addition. Lysosomes below above O/R_{max} or G/R_{max} of *CalipHluor*^{mLy} are shown as >1000µm Ca^{2+} or >pH 6, respectively.

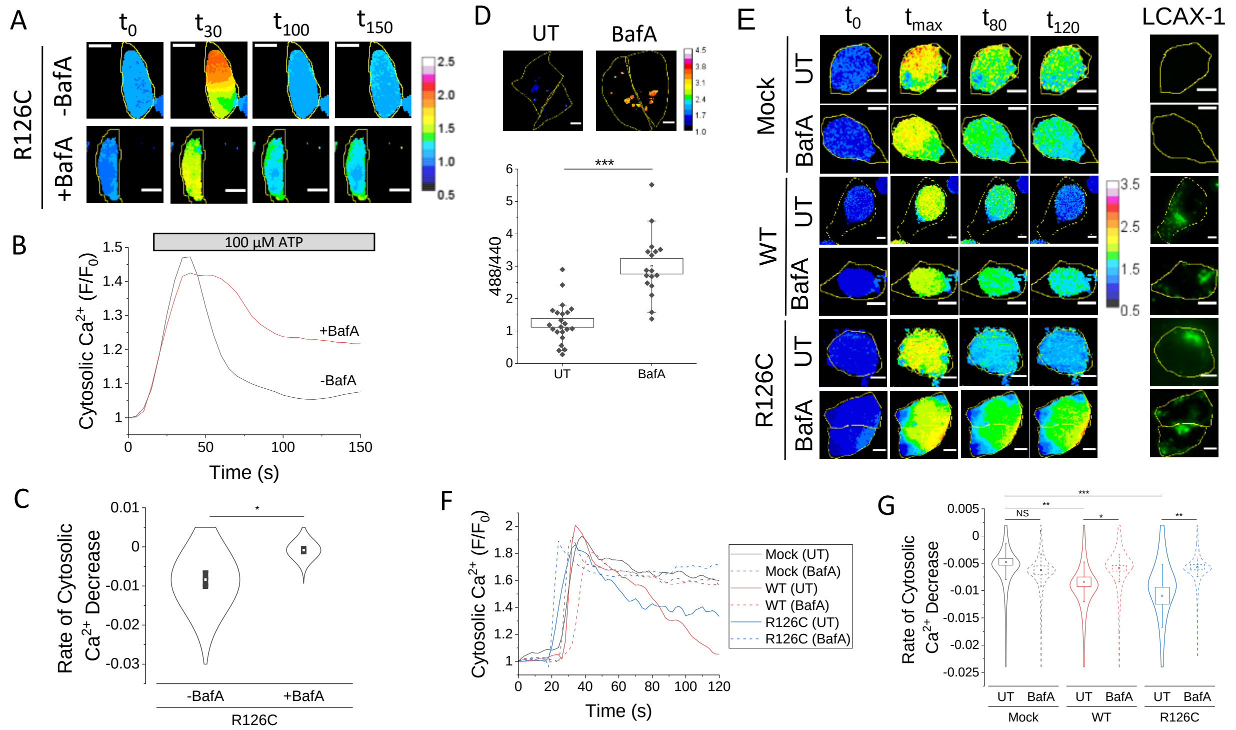

Figure S15. Sensitivity of human LCI to the pH gradient across the lysosomal membrane. (a) Representative 440/488 maps of Fura Red in TMEM165 KO HeLa cells treated with 100 µM ATP transfected with the lysosome-favoring mutant of human LCI-EGFP (R126C). Where indicated, 500 nM bafilomycin A was added 30 minutes prior to and throughout the experiment. Maps shown are at t=0s (prior to ATP treatment), t=30s, t=100s, and t=150s. (b) Representative cytosolic Ca^{2+} traces given by the 440/488 ratio of Fura Red in (a). Curves are normalized to the value at t=0. (c) Slope of the linear fit of the cytosolic Ca^{2+} decrease following maximum 440/488 of Fura Red. (d) Top, representative 488/440 pseudocolored maps of FITC-dextran in COS-7 cells either untreated or treated with 500 nM bafilomycin A for 30 min. Bottom, quantification of 488/440 ratios of individual lysosomes under the indicated conditions. (e) Left, representative 440/488 maps of Fura Red in COS-7 cells treated with 100 µM ATP after mock transfection or transfection with the indicated mutant of human LCI-EGFP. Cells were pre-labelled and treated with 500 nM bafilomycin A prior to imaging and ATP treatment, as

indicated. Maps shown are at t=0s (prior to ATP treatment), maximum 440/488, t=80s, and t=120s. Right, representative GFP images showing transfection of human LCI-EGFP. (f) Representative cytosolic Ca^{2+} levels given by 440/488 intensity of Fura Red in (e). Curves are normalized to the value at t=0. (g) Slope of linear fit of the cytosolic Ca^{2+} decrease following maximum 440/488 of Fura Red. Scale bar 5µm. Boxes and bars indicate the s.e.m. and standard deviation, respectively. Violin plots show the normal distribution. NS, not significant ($P>0.05$); *$P<0.05$; **$P<0.01$; ***$P<0.001$ (one-way ANOVA with Tukey post hoc test).

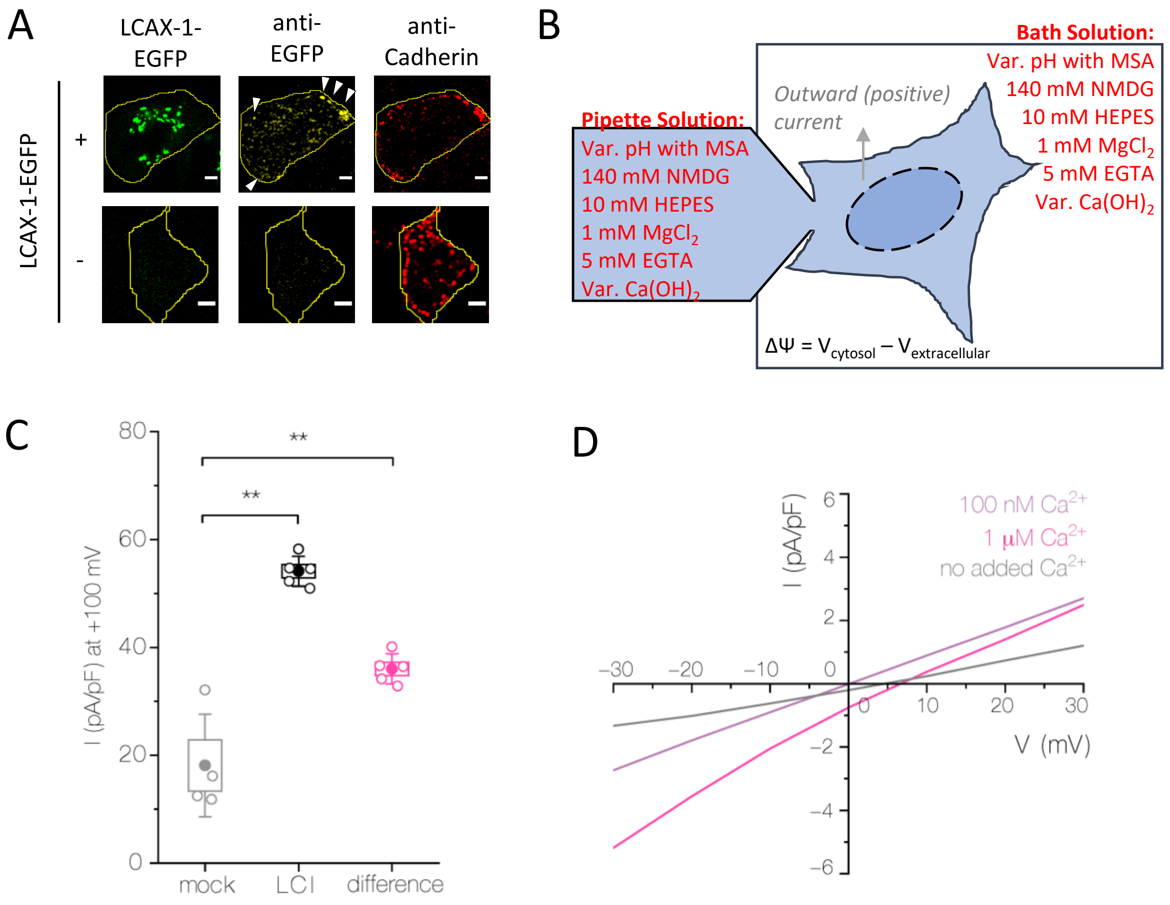

Figure S16. Electrophysiological characterization of human LCI on the plasma membrane. (a) Representative image of human LCI-EGFP-transfected HeLa cells and mock-transfected HeLa cells after fixation and immunostaining for EGFP (yellow) and cadherin (red), without permeabilization. (b) Schematic representation of buffers used for whole-cell patch-clamping, and sign conventions used for current and membrane potential. (c) Current density at +100mV across the plasma membrane of the indicated cell types under the indicated ionic conditions from Fig. 5B. (d) Zoomed-in version of Fig. 5D to show reversal potentials under the indicated conditions. Boxes and bars represent s.e.m. and standard deviation, respectively. **$p<0.01$ (one-way ANOVA with Tukey post hoc test).

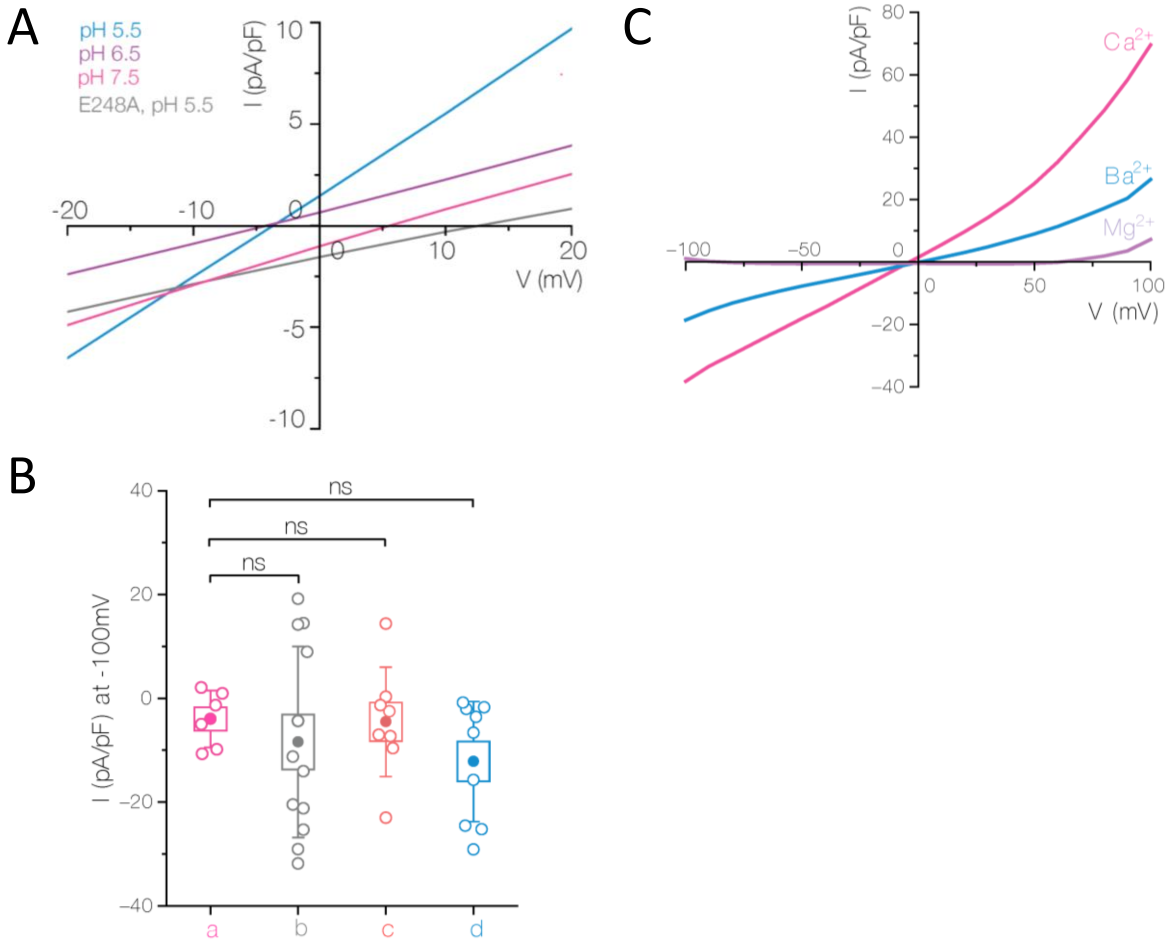

Figure S17. pH dependence of human LCI by whole cell electrophysiology. (a) Zoomed-in version of Fig. 5E to show reversal potentials under the indicated conditions. (b) Current density at -100mV across the plasma membrane of the indicated cell types under the indicated ionic conditions from Fig. 5G. (c) Average current density of mock-subtracted human LCI current in HeLa cells under the indicated conditions, with the indicated divalent cation.

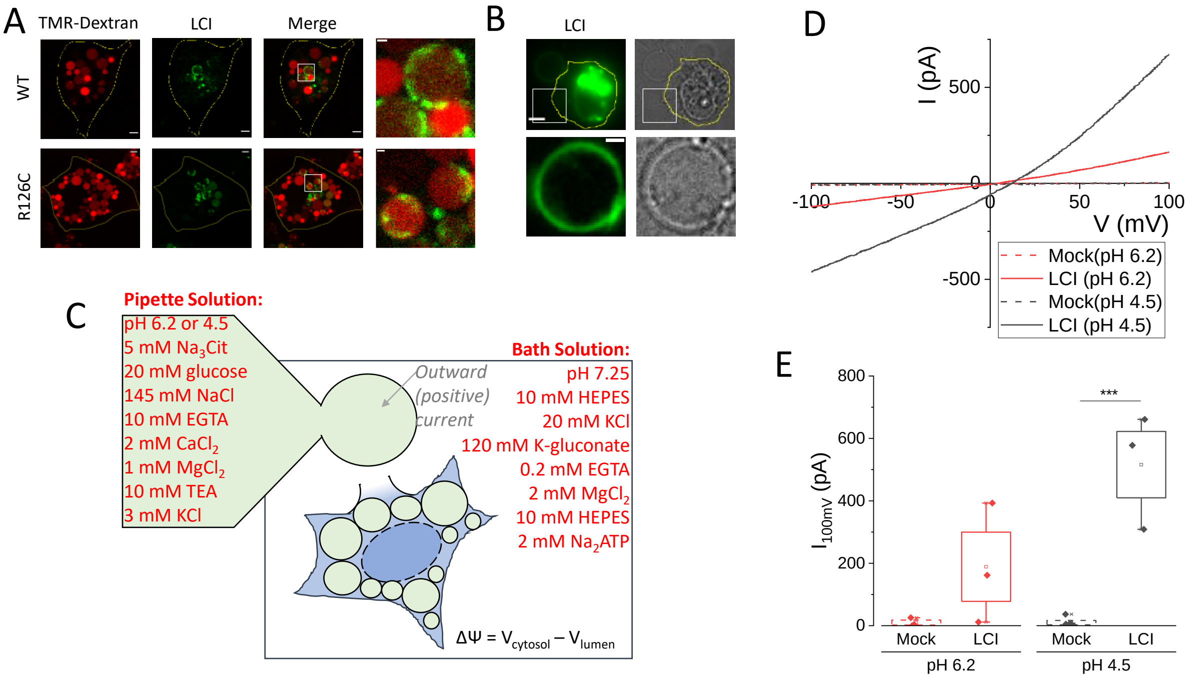

Figure S18. Electrophysiological characterization of human LCI on isolated lysosomes. (a) Representative confocal images of COS-7 cells transiently transfected with WT or R126C human LCI-EGFP (green). Lysosomes were labelled with 1 mg/mL TMR-dextran (red) and swollen with 5µM vacuolin-1 before imaging. (b) Representative fluorescence of human LCI-EGFP (green) on an isolated lysosome of COS-7 cells following overnight treatment with 5 µM vacuolin-1 and cell rupture. (c) Schematic representation of buffers used for lysosome patch-clamping, and sign conventions used for current and membrane potential. (d) Representative IV curves of lysosome patch-clamping using a ramp from -100mV to +100mV with buffers indicated in (c). Lysosomes of mock-transfected HeLa cells (dashed lines) or cells transfected with human LCI-EGFP (solid lines) were patched with pipette solution of pH 6.2 (red) or pH 4.5 (black). (e) Current at +100mV across the lysosome membrane from indicated cells with the indicated lysosomal pH. Scale bar 5µm. Inset scale bar 2µm. Boxes and bars represent s.e.m. and outliers, respectively. *p<0.05.

Table S1.

Genes screened for *cup-5*$^{+/-}$ worm survival rescue, with brood size difference and significance calculated with respect to empty vector (EV).

**Gene**	**Brood Size Difference**	**-log(p-value)**
T28H10.3	212.3568889	1.254384901
C27A7.3	141.1346667	0.755619777
Y54F10AL.1	122.2457778	4.744106592
egl-30	115.0235556	1.469045741
vps-35	114.3568889	3.121612754
sul-3	114.1346667	2.781089912
apd-3	113.6902222	1.986709867
gpa-4	113.6902222	3.655878304
vps-18	110.9124445	1.490814669
catp-5	110.8013333	1.71125926
catp-6	110.1346667	1.500518333
F49B2.6	109.3568889	2.563285232
abt-5	105.3568889	1.563874638
abt-2	104.9124444	1.489412157
pho-14	104.5791111	3.528647045
Y71G12B.23	103.3568889	3.497684353
Y105E8B.9	101.0235556	3.859484617
T27A1.5	101.0235556	1.396960395
C16E9.1	99.96800001	1.653477109
aagr-2	98.02355556	2.618981401
R09F10.1	97.96800001	1.833066059
asah-1	97.46800001	1.173245577
cm-7	96.35688889	2.336553287
unc-108	95.57911114	3.241858374
F22G12.4	94.80133334	2.234028538
ragc-1	89.91244445	1.361245803
C33C12.3	89.13466667	2.997813091
apg-1	84.46800001	2.970542938
F16F9.1	83.13466667	2.190419371
gana-1	83.13466667	0.986737926
nep-2	83.02355556	1.4018297
W03G11.3	82.69022223	1.685793196
asm-2	79.80133334	3.03091558
aph-2	78.13466667	1.121495568
cpz-1	77.91244444	1.688340283
F18A12.8	77.80133334	2.908349875
C18B12.4	77.13466667	1.83082375
B0035.3	77.13466667	1.214323653
asp-3	76.80133334	1.805985669
eat-4	76.35688889	1.732690249
hmit-1.1	75.46800001	2.578440202

apm-3	75.02355556	1.796986763
asm-3	74.24577778	1.523190639
ZK54.1	72.57911112	2.308485448
hsp-70	72.02355556	1.861648144
C38C10.2	70.57911112	1.641794896
C56E6.6	70.24577778	2.885541496
vps-11	69.02355556	1.175622119
apt-9	68.35688889	1.141326101
ced-11	68.13466667	0.704623013
lmp-2	68.02355556	0.449468996
M110.7	67.69022223	1.063067045
F44E5.4	66.91244445	1.129927851
cdf-2	66.35688889	2.725218971
C33G3.4	65.91244445	1.304017205
pcp-1	65.80133334	1.481101529
smf-3	65.24577778	1.413908739
ncr-1	64.57911112	0.755266598
haf-8	64.46800001	1.657782834
vhlu-2	64.02355556	1.015157172
lrp-2	63.46800001	1.715785482
clic-1	62.91244445	0.930238175
lipl-7	62.46800001	1.331036794
F54G2.1	62.35688889	1.333639416
raga-1	61.91244445	2.453983644
nuc-1	61.24577778	1.757458534
aex-1	60.24577778	0.831050663
eak-7	59.91244445	0.632398858
M05B5.4	59.91244444	2.084764907
abt-4	58.46800001	1.257912669
tag-300	58.13466667	0.785166945
cm-6	57.91244445	2.48054339
arr-1	57.69022223	1.86735057
vha-14	57.69022223	0.702913624
Y16B4A.2	57.57911112	1.882167431
C39E9.10	56.02355556	0.819643083
haf-2	55.02355556	0.750986508
tmbi-4	54.91244445	1.679293456
scav-3	54.85688889	1.227311058
cht-1	53.35688889	2.001167993
F44D12.9	52.57911112	0.872154119
C27A7.1	52.35688889	2.196711142
K10C9.3	52.24577778	0.702061596
vha-7	52.13466667	2.271386822
pxn-2	52.13466667	2.096443829
dpf-2	52.02355556	1.157294837
nep-1	51.02355556	1.580778612
hex-1	51.02355556	0.71080953
apb-3	50.46800003	0.829255324

catp-6 P2D12	50.24577778	1.460069613
F13H10.3	49.35688889	0.947524022
chhy-1	49.24577778	2.150389207
drp-1	49.13466667	0.84904707
sulp-5	48.13466667	0.58113876
lipl-5	47.46800001	0.953432696
mrp-7	47.35688889	1.967086212
F02E9.7	47.24577777	1.728542961
Y110A	47.24577767	2.08544863
ppt-1	46.69022223	1.884110841
mrp-5	46.57911112	1.155699658
imp-1	46.35688887	1.688626545
hlh-30	46.30133334	1.596392116
rab-7	46.24577778	2.043680994
rbf-1	44.69022223	0.838397733
ntp-1	43.35688889	1.199145161
vps-41	43.24577778	1.134967524
C29H12.2	43.13466667	0.963805337
R12C12.6	42.57911112	1.144640675
ent-1	42.13466667	1.834503176
nid-1	41.69022223	0.864669048
Y69E1A.5	41.35688889	0.82001007
Y43F4B.7	40.91244445	1.256446446
ZK632.12	38.80133334	0.732614868
tag-120	38.57911112	1.512400118
mlc-1	38.13466667	1.384964145
F22E12.1	37.91244445	1.463588797
pld-1	37.91244445	0.445301821
aagr-1	37.69022223	1.290115829
F42A8.3	37.57911112	0.534211547
mrp-4	37.57911112	1.019544579
T07F10.1	37.35688889	1.220192955
mrp-6	36.57911112	0.863526738
cdf-1	36.57911112	0.665959928
odr-3	35.91244445	1.100725437
vha-12	34.69022223	0.653353994
haf-4	33.80133334	0.787873608
pcp-5	33.63466667	0.987503332
ctns-1	33.46800001	1.083842594
vha-15	33.02355556	0.658190719
Y55D5A.3	32.46800001	1.405752677
sulp-4	31.13466667	1.026442467
F16H11.1	31.02355556	0.387455986
F44E5.5	30.24577778	1.251382942
vps-16	29.57911112	1.001637007
chtl-1	29.57911112	0.661685302
aman-1	28.46800001	0.325465194
arl-8	27.57911112	0.445492982

vhlu-3	27.35688889	0.503347916
xbx-6	26.57911112	0.792095948
lipl-3	26.13466667	0.968611184
C44C1.2	26.02355556	1.057251997
kcc-3	25.80133334	0.351566399
vha-18	25.35688889	0.841584006
lron-8	25.13466686	0.277848285
vps-39	24.69022223	0.591130135
spp-8	24.57911112	0.266858423
ccz-1	24.46800001	0.558713679
tag-130	24.02355556	0.726012845
F57F5.1	23.69022223	1.053545702
trpa-1	22.35688889	0.958664511
R09B5.12	22.35688889	0.570554465
C13C4.5	22.02355556	0.742066139
pxn-1	21.80133334	0.763636959
tag-196	21.46800001	0.772947443
ttm-1	20.35688889	0.503926801
toca-2	19.13466667	0.462554119
sel-12	19.02355556	0.253507056
C33C12.8	18.80133334	0.62419943
lipl-1	17.80133334	0.753820184
hsp-4	17.69022223	0.696292959
sulp-2	16.80133334	0.714723069
jcc01 / kcc-1??	15.46800001	0.248519971
C56A3.8	14.46800001	0.396056749
R04B3.2	14.02355556	0.209973783
tat-2	12.46800001	0.174194958
F42G9.6	12.24577778	0.118017124
K10C2.1	11.57911112	0.31592321
arf-1.2	10.80133334	0.214052324
vha-13	9.91244445	0.368390378
let-363	8.801333439	0.254955754
dnj-14	7.91244445	0.087519381
mig-23	6.579111117	0.120923233
smf-1	6.134666673	0.219877812
mrp-3	6.134666673	0.119370866
cin-3.3	5.91244445	0.214115687
hsp-3	5.134666673	0.06040992
F37H8.5	4.468000006	0.093285049
cpr-2	3.91244445	0.05625215
Y37D8A.8	0.245777784	0.00387559
EV	0	0
Y56A3A.16	-0.309777772	0.006153779
haf-9	-0.309777781	0.003966401
cpr-6	-1.643111105	0.046758532
cin-3.2	-3.754222127	0.072815893
daf-15	-7.643111105	0.144232338

dpf-1	-7.754222216	0.193075056
vps-33.1	-8.08755555	0.181435305
smf-2	-8.865333327	0.10997146
sphk-1	-9.309777772	0.252488153
dhs-22	-10.19866666	0.321736316
cpr-5	-10.75422222	0.244112191
goa-1	-13.53199999	0.41554201
clh-5	-14.53199999	0.213599402
tag-257	-15.53199999	0.534495932
Y53G8AR.7	-16.53199999	0.367649907
rme-8	-16.6431111	0.46463691
C29E4.10	-20.08755555	0.552456358
mrp-2	-20.42088888	0.282638928
cup-5	-26.19866666	0.91547543
arf-3	-31.64311111	0.734212772
mrp-1	-36.42088888	1.339549802
T08A11.1	-39.53199999	1.013321133
hmit-1.3	-50.97644444	1.405485392
unc-32	-51.42088888	2.219450016
unc-93	-58.19866666	1.818691627
prdx-3	-61.86533333	1.787814749
vha-3	-63.64311111	1.689640162
vha-11	-69.36533333	3.04155633
heh-1	-70.42088888	2.719600491
toca-1	-70.73199999	2.86818469
C18H7.1	-71.30977777	2.74764887
gpb-1	-72.53199999	2.914236189
vha-5	-73.30977777	2.972253881
apm-1	-73.86533333	2.69245615
ZK669.2	-77.97644444	2.864406439
cpl-1	-78.19866666	2.823794982
aps-1	-78.69866666	3.008282519
rab-5	-82.08755555	2.861769023
vha-6	-82.78199999	2.862359949
chc-1	-83.19866666	2.89998602
vha-2	-83.64311111	2.865798475
snap-1	-83.75422222	2.882797563
vha-9	-84.08755555	2.876423724
ubq-2	-84.36533333	2.880390266
vha-8	-84.53199999	2.891395193
vha-1	-84.69866666	2.891150092
apb-1	-84.86533333	2.892287095
vha-10	-85.53199999	2.902387253
vha-4	-85.53199999	2.902387253

Table S2.

Sequences of DNA oligos used in this study. D1 and D2 were used for labeling of coelomocyte lysosomes for lysosome size assay and mammalian cell lysosomes for endocytic tracking. C1, C2, and C3 were used to prepare *CalipHluor 2.0*. OG-C1, C2, and C3 were used to prepared *CalipHluor*^{mLy}.

**Sequence Name**	**DNA sequence information**
D1	5'-DBCO-ATC AAC ACT GCA CAC CAG ACA GCA AGA TCC TAT ATA TA-3'
D2	5'-Alexa 647-TA TAT ATA GGA TCT TGC TGT CTG GTG TGC AGT GTT GAT-3'
C1	5'-Amino-ATA ACA CAT AAC ACA TAA CAA AAT ATA TAT CCT AGA ACG ACA GAC AAA CAG TGA GTC-3'
C2	5'-ATTO647-TAT ATT TTG TTA TGT GTT ATG TGT TAT-3'
C3	5'-DBCO-GAC TCA CTG TTT GTC TGT CGT TCT AGG ATA-3'
OG-C1	5'-Oregon Green- ATA ACA CAT AAC ACA TAA CAA AAT ATA TAT CCT AGA ACG ACA GAC AAA CAG TGA GTC-3'

Table S3.

Internal symmetry of human LCI.

Transmembrane Domains	Identity (%)	Similarity (%)
TMD1-TMD4	28.0	76.0
TMD2-TMD5	35.0	65.0
TMD3-TMD6	21.4	64.0

Table S4.

Internal symmetry of vcx-1.

**Transmembrane Domains**	**Identity (%)**	**Similarity (%)**
TMD1-TMD6	31.2	75.0
TMD2-TMD7	35.0	70.0
TMD3-TMD8	31.6	57.9
TMD4-TMD9	26.7	66.7
TMD5-TMD10	38.5	84.6

Table S5.

Templates used for homology-based modelling of human LCI.

**LCI Domain**	**Template Protein**	**Organism**	**Identity (%)**	**Similarity (%)**
TMD-Reg	C-C chemokine receptor type 9	*Homo sapiens*	71.40	78.60
TMD1	VCX1 M2b	*Saccharomyces cerivisiae*	36.36	54.54
TMD2	Monovalent cation-H^+ antiporter	*Pyrococcus furiosus*	85.70	85.70
TMD3	Two-pore calcium channel protein 2	*Homo sapiens*	50.00	70.00
TMD4	Calcium permeable stress-gated cation channel 1	*Arabidopsis thaliana*	61.50	76.90
TMD5	Calcium homeostasis modulator protein 2	*Homo sapiens*	44.40	55.60
TMD6	Neimann-Pick C1 protein	*Homo sapiens*	63.20	78.90

Table S6.

Reversal potentials expected for exchanger with pipette buffer of pH 7.5 and 1µM Ca and bath buffer of 100µM Ca and indicated pH.

Bath pH	Stoichiometry of Ca^{2+}:H^{+}	Reversal potential
5.5	1:1	-5.20E-14mV
	1:2	∞
	1:3	234mV
	1:4	175.5mV
	1:5	156mV
	2:1	39mV
	2:3	-117mV
	2:5	351mV
	3:1	46.8mV
	3:2	29.25mV
	3:4	-58.5mV
	3:5	-234mV
6.5	1:1	58.5mV
	1:2	∞
	1:3	58.5mV
	1:4	58.5mV
	1:5	58.5mV
	2:1	58.5mV
	2:3	58.5mV
	2:5	58.5mV
	3:1	58.5mV
	3:2	58.5mV
	3:4	58.5mV
	3:5	58.5mV
7.5	1:1	117mV
	1:2	∞

	1:3	-117mV
	1:4	-58.5mV
	1:5	-39mV
	2:1	78mV
	2:3	234mV
	2:5	-234mV
	3:1	70.2mV
	3:2	87.75mV
	3:4	175.5mV
	3:5	351mV

REFERENCES AND NOTES

1. A. Raffaello, C. Mammucari, G. Gherardi, R. Rizzuto, Calcium at the center of cell signaling: Interplay between endoplasmic reticulum, mitochondria, and lysosomes. *Trends Biochem. Sci.* **41**, 1035–1049 (2016).

2. A. Verkhratsky, Physiology and pathophysiology of the calcium store in the endoplasmic reticulum of neurons. *Physiol. Rev.* **85**, 201–279 (2005).

3. S. Marchi, S. Patergnani, S. Missiroli, G. Morciano, A. Rimessi, M. R. Wieckowski, C. Giorgi, P. Pinton, Mitochondrial and endoplasmic reticulum calcium homeostasis and cell death. *Cell Calcium*. **69**, 62–72 (2018).

4. M. Yamasaki-Mann, I. Parker, Enhanced ER Ca^{2+} store filling by overexpression of SERCA2b promotes IP_3-evoked puffs. *Cell Calcium* **50**, 36–41 (2011).

5. A. Brambila, D. M. Robinson, Observing compensatory effects of RyR2, NCX and SERCA down-regulation in neonatal cardiomyocytes using siRNA. *FASEB J.* **29**, 711.29 (2015).

6. S. Sommakia, P. R. Houlihan, S. S. Deane, J. A. Simcox, N. S. Torres, M.-Y. Jeong, D. R. Winge, C. J. Villanueva, D. Chaudhuri, Mitochondrial cardiomyopathies feature increased uptake and diminished efflux of mitochondrial calcium. *J. Mol. Cell. Cardiol.* **113**, 22–32 (2017).

7. D. De Stefani, A. Raffaello, E. Teardo, I. Szabò, R. Rizzuto, A forty-kilodalton protein of the inner membrane is the mitochondrial calcium uniporter. *Nature* **476**, 336–340 (2011).

8. J. M. Baughman, F. Perocchi, H. S. Girgis, M. Plovanich, C. A. Belcher-Timme, Y. Sancak, X. R. Bao, L. Strittmatter, O. Goldberger, R. L. Bogorad, V. Koteliansky, V. K. Mootha, Integrative genomics identifies MCU as an essential component of the mitochondrial calcium uniporter. *Nature* **476**, 341–345 (2011).

9. D. Chaudhuri, Y. Sancak, V. K. Mootha, D. E. Clapham, MCU encodes the pore conducting mitochondrial calcium currents. *eLife* **2**, e00704 (2013).

10. H. C. Lee, R. Aarhus, A derivative of NADP mobilizes calcium stores insensitive to inositol trisphosphate and cyclic ADP-ribose. *J. Biol. Chem.* **270**, 2152–2157 (1995).

11. B. S. Kilpatrick, E. R. Eden, A. H. Schapira, C. E. Futter, S. Patel, Direct mobilisation of lysosomal Ca^{2+} triggers complex Ca^{2+} signals. *J. Cell Sci.* **126**, 60–66 (2013).

12. M. Brini, E. Carafoli, The plasma membrane Ca^{2+} ATPase and the plasma membrane sodium calcium exchanger cooperate in the regulation of cell calcium. *Cold Spring Harb. Perspect. Biol.* **3,** a004168 (2011).

13. J. K. Pittman, Vacuolar Ca^{2+} uptake. *Cell Calcium* **50**, 139–146 (2011).

14. K. A. Christensen, J. T. Myers, J. A. Swanson, pH-dependent regulation of lysosomal calcium in macrophages. *J. Cell Sci.* **115**, 599–607 (2002).

15. S. A. Hilden, N. E. Madias, H^+/Ca^{2+} exchange in rabbit renal cortical endosomes. *J. Membr. Biol.* **112**, 131–138 (1989).

16. C. I. López-Sanjurjo, S. C. Tovey, D. L. Prole, C. W. Taylor, Lysosomes shape Ins(1,4,5)P_3-evoked Ca^{2+} signals by selectively sequestering Ca^{2+} released from the endoplasmic reticulum. *J. Cell Sci.* **126**, 289–300 (2013).

17. N. Narayanaswamy, K. Chakraborty, A. Saminathan, E. Zeichner, K. Leung, J. Devany, Y. Krishnan, A pH-correctable, DNA-based fluorescent reporter for organellar calcium. *Nat. Methods* **16**, 95–102 (2019).

18. M. Melchionda, J. K. Pittman, R. Mayor, S. Patel, Ca^{2+}/H^+ exchange by acidic organelles regulates cell migration in vivo. *J. Cell Biol.* **212**, 803–813 (2016).

19. L. Schaheen, G. Patton, H. Fares, Suppression of thecup-5mucolipidosis type IV-related lysosomal dysfunction by the inactivation of an ABC transporter inC. elegans. *Development* **133**, 3939–3948 (2006).

20. H. Fares, I. Greenwald, Genetic analysis of endocytosis in Caenorhabditis elegans: Coelomocyte uptake defective mutants. *Genetics* **159**, 133–145 (2001).

21. D. Demaegd, A.-S. Colinet, A. Deschamps, P. Morsomme, Molecular evolution of a novel family of putative calcium transporters. *PLOS ONE* **9**, e100851 (2014).

22. E. Dulary, S. Potelle, D. Legrand, F. Foulquier, TMEM165 deficiencies in congenital disorders of glycosylation type II (CDG-II): Clues and evidences for roles of the protein in Golgi functions and ion homeostasis. *Tissue Cell* **49**, 150–156 (2017).

23. F. Foulquier, M. Amyere, J. Jaeken, R. Zeevaert, E. Schollen, V. Race, R. Bammens, W. Morelle, C. Rosnoblet, D. Legrand, D. Demaegd, N. Buist, D. Cheillan, N. Guffon, P. Morsomme, W. Annaert, H. H. Freeze, E. Van Schaftingen, M. Vikkula, G. Matthijs, TMEM165 deficiency causes a congenital disorder of glycosylation. *Am. J. Hum. Genet.* **91**, 15–26 (2012).

24. C. Rosnoblet, D. Legrand, D. Demaegd, H. Hacine-Gherbi, G. de Bettignies, R. Bammens, C. Borrego, S. Duvet, P. Morsomme, G. Matthijs, F. Foulquier, Impact of disease-causing mutations on TMEM165 subcellular localization, a recently identified protein involved in CDG-II. *Hum. Mol. Genet.* **22**, 2914–2928 (2013).

25. R. Zeevaert, F. de Zegher, L. Sturiale, D. Garozzo, M. Smet, M. Moens, G. Matthijs, J. Jaeken, Bone dysplasia as a key feature in three patients with a novel congenital disorder of glycosylation (CDG) type ii due to a deep intronic splice mutation in TMEM165. *JIMD Rep.* **8**, 145–152 (2013).

26. M. V. Matz, A. F. Fradkov, Y. A. Labas, A. P. Savitsky, A. G. Zaraisky, M. L. Markelov, S. A. Lukyanov, Fluorescent proteins from nonbioluminescent Anthozoa species. *Nat. Biotechnol.* **17**, 969–973 (1999).

27. M. Zajac, S. Modi, Y. Krishnan, The evolution of organellar calcium mapping technologies. *Cell Calcium* **108**, 102658 (2022).

28. D. Demaegd, F. Foulquier, A.-S. Colinet, L. Gremillon, D. Legrand, P. Mariot, E. Peiter, E. Van Schaftingen, G. Matthijs, P. Morsomme, Newly characterized Golgi-localized family of proteins is involved in calcium and pH homeostasis in yeast and human cells. *Proc. Natl. Acad. Sci. U.S.A.* **110**, 6859–6864 (2013).

29. A. B. Waight, B. P. Pedersen, A. Schlessinger, M. Bonomi, B. H. Chau, Z. Roe-Zurz, A. J. Risenmay, A. Sali, R. M. Stroud, Structural basis for alternating access of a eukaryotic calcium/proton exchanger. *Nature* **499**, 107–110 (2013).

30. A. Miseta, R. Kellermayer, D. P. Aiello, L. Fu, D. M. Bedwell, The vacuolar Ca^{2+} /H^{+} exchanger Vcx1p/Hum1p tightly controls cytosolic Ca^{2+} levels in *S. cerevisiae*. *FEBS Lett.* **451**, 132–136 (1999).

31. J. Stribny, L. Thines, A. Deschamps, P. Goffin, P. Morsomme, The human Golgi protein TMEM165 transports calcium and manganese in yeast and bacterial cells. *J. Biol. Chem.* **295**, 3865–3874 (2020).

32. N. A. Snyder, M. V. Palmer, T. A. Reinhardt, K. W. Cunningham, Milk biosynthesis requires the Golgi cation exchanger TMEM165. *J. Biol. Chem.* **294**, 3181–3191 (2019).

33. X. Cai, J. Lytton, The cation/Ca^{2+} exchanger superfamily: Phylogenetic analysis and structural implications. *Mol. Biol. Evol.* **21**, 1692–1703 (2004).

34. S. Schulte Althoff, M. Grüneberg, J. Reunert, J. H. Park, S. Rust, C. Mühlhausen, Y. Wada, R. Santer, T. Marquardt, TMEM165 deficiency: Postnatal changes in glycosylation. *JIMD Rep.* **26**, 21–29 (2016).

35. K. D. Hirschi, R. G. Zhen, K. W. Cunningham, P. A. Rea, G. R. Fink, CAX1, an H+/Ca2+ antiporter from Arabidopsis. *Proc. Natl. Acad. Sci. U.S.A.* **93**, 8782–8786 (1996).

36. S.-L. Tan, M. Barri, P. Atakpa-Adaji, C. W. Taylor, E. St John Smith, R. D. Murrell-Lagnado, P2X4 receptors mediate Ca^{2+} release from lysosomes in response to stimulation of P2X7 and H1 histamine receptors. *Int. J. Mol. Sci.* **22,** 10492 (2021).

37. K. Leung, K. Chakraborty, A. Saminathan, Y. Krishnan, A DNA nanomachine chemically resolves lysosomes in live cells. *Nat. Nanotechnol.* **14**, 176–183 (2019).

38. P. Anees, A. Saminathan, E. R. Rozmus, A. Di, A. B. Malik, B. P. Delisle, Y. Krishnan, Detecting organelle-specific activity of potassium channels with a DNA nanodevice. *Nat. Biotechnol.* (2023).

39. P. Pihán, F. Lisbona, J. Borgonovo, S. Edwards-Jorquera, P. Nunes-Hasler, K. Castillo, O. Kepp, H. Urra, S. Saarnio, H. Vihinen, A. Carreras-Sureda, S. Forveille, A. Sauvat, D. De Giorgis, A. Pupo, D. A. Rodríguez, G. Quarato, A. Sagredo, F. Lourido, A. Letai, R. Latorre, G. Kroemer, N. Demaurex, E. Jokitalo, M. L. Concha, Á. Glavic, D. R. Green, C. Hetz, Control of lysosomal-mediated cell death by the pH-dependent calcium channel RECS1. *Sci. Adv.* **7**, eabe5469 (2021).

40. M. Uhlén, L. Fagerberg, B. M. Hallström, C. Lindskog, P. Oksvold, A. Mardinoglu, Å. Sivertsson, C. Kampf, E. Sjöstedt, A. Asplund, I. Olsson, K. Edlund, E. Lundberg, S. Navani, C. A. Szigyarto, J. Odeberg, D. Djureinovic, J. O. Takanen, S. Hober, T. Alm, P. H. Edqvist, H. Berling, H. Tegel, J. Mulder, J. Rockberg, P. Nilsson, J. M. Schwenk, M. Hamsten, K. von Feilitzen, M. Forsberg, L. Persson, F. Johansson, M. Zwahlen, G. von Heijne, J. Nielsen, F. Pontén, Tissue-based map of the human proteome. *Science* **347**, 1260419 (2015).

41. Y. Kirichok, G. Krapivinsky, D. E. Clapham, The mitochondrial calcium uniporter is a highly selective ion channel. *Nature* **427**, 360–364 (2004).

42. A. Tinker, A. J. Williams, Divalent cation conduction in the ryanodine receptor channel of sheep cardiac muscle sarcoplasmic reticulum. *J. Gen. Physiol.* **100**, 479–493 (1992).

43. B. D. Delgado, S. B. Long, Mechanisms of ion selectivity and throughput in the mitochondrial calcium uniporter. *Sci. Adv.* **8**, eade1516 (2022).

44. V. Garg, J. Suzuki, I. Paranjpe, T. Unsulangi, L. Boyman, L. S. Milescu, W. J. Lederer, Y. Kirichok, The mechanism of MICU-dependent gating of the mitochondrial Ca2+uniporter. *eLife* **10,** e69312 (2021).

45. X. Dong, D. Shen, X. Wang, T. Dawson, X. Li, Q. Zhang, X. Cheng, Y. Zhang, L. S. Weisman, M. Delling, H. Xu, PI(3,5)P_2 controls membrane trafficking by direct activation of mucolipin Ca^{2+} release channels in the endolysosome. *Nat. Commun.* **1**, 38 (2010).

46. R. Bammens, N. Mehta, V. Race, F. Foulquier, J. Jaeken, M. Tiemeyer, R. Steet, G. Matthijs, H. Flanagan-Steet, Abnormal cartilage development and altered N-glycosylation in Tmem165-deficient zebrafish mirrors the phenotypes associated with TMEM165-CDG. *Glycobiology* **25**, 669–682 (2015).

47. P. Murali, B. P. Johnson, Z. Lu, L. Climer, D. A. Scott, F. Foulquier, G. Oprea-Ilies, V. Lupashin, R. R. Drake, K. L. Abbott, Novel role for the Golgi membrane protein TMEM165 in control of migration and invasion for breast carcinoma. *Oncotarget* **11**, 2747–2762 (2020).

48. S. Khan, M. Sbeity, F. Foulquier, L. Barré, M. Ouzzine, TMEM165 a new player in proteoglycan synthesis: Loss of TMEM165 impairs elongation of chondroitin- and heparan-sulfate glycosaminoglycan chains of proteoglycans and triggers early chondrocyte differentiation and hypertrophy. *Cell Death Dis.* **13**, 11 (2022).

49. B. Suresh, A. Saminathan, K. Chakraborty, C. Cui, L. Becker, Y. Krishnan, Tubular lysosomes harbor active ion gradients and poise macrophages for phagocytosis. BioRxiv 2020.12.05.413229. https://doi.org/10.1101/2020.12.05.413229

50. S. Brenner, The genetics of Caenorhabditis elegans. *Genetics* **77**, 71–94 (1974).

51. V. Au, E. Li-Leger, G. Raymant, S. Flibotte, G. Chen, K. Martin, L. Fernando, C. Doell, F. I. Rosell, S. Wang, M. L. Edgley, A. E. Rougvie, H. Hutter, D. G. Moerman, CRISPR/Cas9 methodology for the generation of knockout deletions in Caenorhabditis elegans. *G3*. **9**, 135–144 (2019).

52. R. S. Kamath, J. Ahringer, Genome-wide RNAi screening in Caenorhabditis elegans. *Methods* **30**, 313–321 (2003).

53. K. Chakraborty, K. Leung, Y. Krishnan, High lumenal chloride in the lysosome is critical for lysosome function. *eLife* **6**, e28862 (2017).

54. K. Dan, A. T. Veetil, K. Chakraborty, Y. Krishnan, DNA nanodevices map enzymatic activity in organelles. *Nat. Nanotechnol.* **14**, 252–259 (2019).

55. S. Surana, A. R. Shenoy, Y. Krishnan, Designing DNA nanodevices for compatibility with the immune system of higher organisms. *Nat. Nanotechnol.* **10**, 741–747 (2015).

56. J. Zou, K. Mitra, P. Anees, D. Oettinger, J. R. Ramirez, A. T. Veetil, P. D. Gupta, R. Rao, J. J. Smith, P. Kratsios, Y. Krishnan, A DNA nanodevice for mapping sodium at single-organelle resolution. *Nat. Biotechnol.* (2023).

57. C. Grimm, J. Vierock, P. Hegemann, J. Wietek, Whole-cell patch-clamp recordings for electrophysiological determination of ion selectivity in channelrhodopsins. *J. Vis. Exp.* (2017).

58. H. Dong, J. Dunn, J. Lytton, Stoichiometry of the Cardiac Na^+/Ca^{2+} exchanger NCX1.1 measured in transfected HEK cells. *Biophys. J.* **82**, 1943–1952 (2002).

59. X.-P. Dong, X. Cheng, E. Mills, M. Delling, F. Wang, T. Kurz, H. Xu, The type IV mucolipidosis-associated protein TRPML1 is an endolysosomal iron release channel. *Nature* **455**, 992–996 (2008).

60. K. W. Cunningham, G. R. Fink, Calcineurin-dependent growth control in Saccharomyces cerevisiae mutants lacking PMC1, a homolog of plasma membrane Ca^{2+} ATPases. *J. Cell Biol.* **124**, 351–363 (1994).

61. K. G. Sharma, R. Kaur, A. K. Bachhawat, The glutathione-mediated detoxification pathway in yeast: An analysis using the red pigment that accumulates in certain adenine biosynthetic mutants of yeasts reveals the involvement of novel genes. *Arch. Microbiol.* **180**, 108–117 (2003).

62. T. R. Amen, E. V. Mikhailova, V. V. Alenin, A. V. Artyomov, P. A. Dementyev, M. A. Khodorkovskii, T. O. Artamonova, I. M. Kuznetsova, T. R. Soidla, O. V. Nevzglyadova, Structural and functional characteristics of various forms of red pigment of yeast *Saccharomyces cerevisiae* and its synthetic analog. *Cell Tissue Biol.* **7**, 86–94 (2013).

63. H. Tournu, J. Carroll, B. Latimer, A.-M. Dragoi, S. Dykes, J. Cardelli, T. L. Peters, K. E. Eberle, G. E. Palmer, Identification of small molecules that disrupt vacuolar function in the pathogen Candida albicans. *PLOS ONE* **12**, e0171145 (2017).
